# HIV-exposure, early life feeding practices and delivery mode impacts on faecal bacterial profiles in a South African birth cohort

**DOI:** 10.1038/s41598-018-22244-6

**Published:** 2018-03-22

**Authors:** Shantelle Claassen-Weitz, Sugnet Gardner-Lubbe, Paul Nicol, Gerrit Botha, Stephanie Mounaud, Jyoti Shankar, William C Nierman, Nicola Mulder, Shrish Budree, Heather J. Zar, Mark P. Nicol, Mamadou Kaba

**Affiliations:** 1 Division of Medical Microbiology, Department of Pathology, Faculty of Health Sciences, University of Cape Town, Cape Town, South Africa; 2 Department of Statistics and Actuarial Science, Faculty of Economic and Management Sciences, Stellenbosch University, Stellenbosch, South Africa; 3 Computational Biology Group and H3ABioNet, Department of Integrative Biomedical Sciences, University of Cape Town, Cape Town, South Africa; 4 J. Craig Venter Institute, Rockville, Maryland United States of America; 5 Department of Paediatrics and Child Health, Red Cross War Memorial Children’s Hospital, Cape Town, South Africa; 6 OpenBiome, Somerville, Massachusetts, United States of America; 7 SAMRC Unit on Child & Adolescent Health, University of Cape Town, Cape Town, South Africa; 8 Institute of Infectious Disease and Molecular Medicine, Faculty of Health Sciences, University of Cape Town, Cape Town, South Africa; 9 National Health Laboratory Service of South Africa, Groote Schuur Hospital, Cape Town, South Africa

## Abstract

There are limited data on meconium and faecal bacterial profiles from African infants and their mothers. We characterized faecal bacterial communities of infants and mothers participating in a South African birth cohort. Stool and meconium specimens were collected from 90 mothers and 107 infants at birth, and from a subset of 72 and 36 infants at 4–12 and 20–28 weeks of age, respectively. HIV-unexposed infants were primarily exclusively breastfed at 4–12 (49%, 26/53) and 20–28 weeks (62%, 16/26). In contrast, HIV-exposed infants were primarily exclusively formula fed at 4–12 (53%; 10/19) and 20–28 weeks (70%, 7/10). Analysis (of the bacterial 16S rRNA gene sequences of the V4 hypervariable region) of the 90 mother-infant pairs showed that meconium bacterial profiles [dominated by Proteobacteria (89%)] were distinct from those of maternal faeces [dominated by Firmicutes (66%) and Actinobacteria (15%)]. Actinobacteria predominated at 4–12 (65%) and 20–28 (50%) weeks. HIV-exposed infants had significantly higher faecal bacterial diversities at both 4–12 (p = 0.026) and 20–28 weeks (p = 0.002). HIV-exposed infants had lower proportions of *Bifidobacterium* (p = 0.010) at 4–12 weeks. Maternal faecal bacterial profiles were influenced by HIV status, feeding practices and mode of delivery. Further longitudinal studies are required to better understand how these variables influence infant and maternal faecal bacterial composition.

## Introduction

Early life bacterial colonization of the gastro-intestinal tract (GIT) has been reported to occur at birth^1^. However, the detection of bacteria in-utero^2–6^, and in the newborn’s meconium^7–9^, suggests foetal colonization^10–12^. Following in-utero colonization^13^; mode of delivery^14–17^, feeding practices^17–19^, weaning^20–22^, use of antibiotics^23^ and latitude^24,25^ are all involved in shaping early life faecal bacterial profiles. Of these exposures, mode of feeding has been shown to greatly influence the composition, diversity and function of early life microbiota^26–28^, which persists even after the introduction of solid food^28^. Notably, breastfeeding has been associated with a number of health benefits for the infant^29,30^. Despite these findings, fewer HIV-infected South African women intend to exclusively breastfeed^31^, regardless of World Health Organization (WHO) recommendations in the context of HIV infection^32^.

The purpose of this study was to characterize meconium and early life faecal bacterial profiles of HIV-exposed and -unexposed infants enrolled in a South African birth cohort study, the Drakenstein Child Health Study (DCHS). To our knowledge, no studies have reported on the meconium bacterial profiles of African newborns, despite African children having different GIT bacterial communities compared to those from high income countries^33,34^. We also aimed to identify key determinants of infant meconium and faecal bacterial profiles in HIV-exposed and -unexposed infants. We further aimed to compare infant meconium with maternal faecal bacterial profiles collected at the time of delivery in order to address the role of maternal GIT microbiota in in-utero colonization of the infant GIT in an African cohort.

## Methodology

### Ethics approval and consent to participate

This study (585/2015), and the DCHS (401/2009), received ethical approval from the Faculty of Health Sciences, Human Research Ethics Committee (HREC) of the University of Cape Town, South Africa. All experiments were performed in accordance with relevant guidelines and regulations. Mothers provided informed, written consent for enrolment of their infants at the time of delivery and annually.

### Study participants

We investigated faecal bacterial profiles from mother-infant pairs enrolled in the DCHS, a birth cohort study investigating the early life determinants of child health in a peri-urban area 60 kilometres from Cape Town, South Africa^35^. Enrolment of pregnant women took place at 20–28 weeks of gestation during antenatal clinic visits in two low socioeconomic communities, TC Newman (primarily a mixed ancestry population) and Mbekweni (primarily a black African population). The selection of participants included in this pilot study was based on the availability of faecal specimens from mothers and infants at the time of delivery and from infants at follow-up visits. The DCHS, collected faecal specimens at six-monthly intervals from all infants, with a convenience subset from whom faecal specimens were collected at monthly visits during the first year of life^35^. Antenatal and early life tobacco smoke exposure was measured via urine cotinine testing^36^ and HIV-exposed infants were screened for HIV infection at 6–10 weeks and at 9 months, or if clinically indicated^37^.

### Specimen collection

Study staff collected faecal specimens (using sterile spatulas and faecal screw-cap containers) from mothers and infants (meconium) at birth. Faecal specimens were also collected longitudinally from infants during scheduled study visits^35^. For this study, we selected faecal specimens collected at 4 to 12 weeks and 20 to 28 weeks of age. Intervals were selected to include the maximum number of longitudinal sample sets available at the time of study. In the event where meconium specimens were not collected prior to discharge, mothers with freezers at home were encouraged to collect and store their infants’ first faecal discharge at −20 °C. Transport of faecal specimens was performed under controlled conditions using ice boxes. Upon arrival at the laboratory, faecal specimens were stored at −80 °C until further processing.

### Nucleic acid extraction

We extracted nucleic acid from faecal specimens (approximately 50 mg) using the QIAsymphony DSP Virus/Pathogen Mini Kit^®^ (Qiagen GmbH, Hilden, Germany) as previously described^38^. Extracted DNA was quantified using the Qubit^®^ 2.0 Fluorometer (Invitrogen^TM^, CA, USA) together with the Qubit™ dsDNA HS Assay Kit (Invitrogen^TM^, CA, USA).

### 16S ribosomal ribonucleic acid (rRNA) amplicon library preparation

We performed two polymerase chain reactions (PCRs) using primers targeting the V4 hypervariable region of the 16S rRNA gene, with minor modifications^39^. In the first PCR reaction, we used modified-F515 (5′ - GTGCCAGCHGCYGCGGT - 3′) and R806 (5′ - GGACTACNNGGGTWTCTAAT - 3′ to amplify the V4 region of the bacterial 16S rRNA gene. The reaction consisted of 12.5 μl of 2X MyTaq^TM^ HS Mix (Bioline, MA, USA), 2 μl forward and 2 μl reverse primer (each at a10 μM initial concentration), 0.75 μl of dimethyl sulfoxide (catalogue no D2650, Sigma-Aldrich^®^, MO, USA) and 4 μl template, made up to a final volume of 25.25 μl using PCR-grade water (Thermo Fisher Scientific Inc., MA, USA). Cycling conditions included a denaturation step at 95 °C for 3 min, an amplification step at 95 °C for 30 sec, 50 °C for 30 sec and 72 °C for 1 sec (proceeding for 10 cycles); and a final extension step at 72 °C for 5 min. In the second PCR, we used 4 μl of the amplicon from the first PCR to add adapters, barcodes, 12–15 staggered nucleotides (NNNNNNNNNNNN) and priming regions. During this second PCR, we used composite primers F515-composite (5′ - AATGATACGGCGACCACCGAGATCTACACTCTTTCCCTACACGACGCTCTTCCGATCTNNNNNNNNNNGTGCCAGCHGCYGCGGT - 3′) and R806-composite (5′ - CAAGCAGAAGACGGCATACGAGATACGAGACTGATTGTGACTGGAGTTCAGACGTGTGCTCTTCCGATCTNNNNNNNNNNNNGGACTACNNGGGTWTCTAAT - 3′) . The two-step amplification approach was performed to reduce the risk of non-specific binding when using adapters/sequencing primers of more than 100bp^40^. Staggered random nucleotides were incorporated to improve cluster identity and imaging in more diverse sample types^40^. Golay barcodes (12 bases underlined in the reverse primer R806-composite)^39^ served to multiplex samples. The only change made to the cycling conditions in the second PCR was the addition of 20 cycles during the amplification step. Amplicons were cleaned using the Agencourt^®^ AMPure^®^ XP PCR Purification kit (Beckman Coulter, CA, USA). Slight modifications to the manufacturer’s protocol included the use of a 0.65:1 ratio of Agencourt AMPure XP solution to PCR products in step 2. PCR products were verified and annotated by agarose gel electrophoresis. We quantified amplicons using the Quanti-iT^TM^ PicoGreen^®^ dsDNA Reagent (Life Technologies, CA, USA) on the Infinite M1000 Pro® microplate reader (Tecan Group Ltd., Grödig, Austria) equipped with Tecan i-Control^TM^ 1.7 software. Following pooling of amplicons at 100 ng, we quantified and purified the library using the Nanodrop ND 1000 (Thermo Fisher Scientific Inc., MA, USA) equipped with ND-1000 3.7.1 software and Agencourt AMPure XP solution (at a 1:1 ratio), respectively. The pooled 16S library was excised following agarose gel electrophoresis, and purified using the QIAquick Gel Extraction kit (QIAgen, MA, USA). Minor modifications to the manufacturer’s protocol included incubation of the sample at 37 °C for 5 min at step 10 and heating of the elution buffer, Tris-EDTA buffer (pH 8.0), between 60 and 70 °C at step 13.

### 16S ribosomal RNA gene sequencing

The 16S library from faecal specimens was sequenced using the MiSeq Reagent Kit v3, 600 cycles (Illumina, CA, USA). Sequencing controls included two Human Microbiome Project mock community controls (HM-782D and HM-783D) (BEI Resources, ATCC, VA, USA) and two no-template water controls. In addition, to serve as inter-run reproducibility measures, we randomly selected nucleic acid extracts from 17 faecal specimens within our cohort to be amplified and sequenced in duplicate. The KAPA qPCR quantification kit (KAPA Biosystems, MA, USA), as well as the Agilent DNA 1000 kit (Agilent Technologies, CA, USA) were used to quantify and size the library. Using these metrics, we diluted the library to 4 nM using Buffer EB (Qiagen, Hilden, Germany), and denaturatured and neutralized the library using 0.2 N NaOH and hybridization buffer (HT1). We prepared a final library dilution at 4 pM to which the internal control (15% PhiX) was added at 4 pM. The denatured library was loaded to the Illumina® MiSeq^TM^ platform as per manufacturer’s instructions^41^.

### Bioinformatics pipeline

We assessed sequencing quality of FASTQ files using Fastqc^42^ and SolexaQA^43^. We then merged forward and reverse sequences using USEARCH7 fastq_mergepairs (fastq_maxdiffs set to 3)^44^, followed by quality filtering using USEARCH7 fastq_filter (sequences truncated to 250 and fastq_maxee set to 0.1). Sequences from no-template water controls were aligned to biological sample sequences using USEARCH7 usearch-global in order to remove potential contaminants. We matched each unique sequence from the two no-template controls to reads present in our biological samples. The average number of reads calculated from the two no-template controls were removed from biological samples in the event that reads matched at 100% similarity. USEARCH sortbysize allowed for dereplication and selection of sequences occurring more than twice. We clustered sequences into operational taxonomic units (OTUs) using USEARCH7 cluster_otus (with a clustering radius of 3). The ChimeraSlayer reference database^45^ and the USEARCH7 uchime_ref tool were used to remove chimeras. OTU counts were obtained using USEARCH7 usearch-global. Further processing of data was performed using the Quantitative Insights Into Microbial Ecology (QIIME 1.7.0) suite of software tools^46^. We assigned taxonomy to representative reads, selecting SILVA^47^ as the reference database and a 97% sequence similarity, using the RDP classifier method implemented through the assign_taxonomy.py script in QIIME^48^. Sequences were aligned (align_seqs.py) at 97% similarity using the PYNAST algorithm and filtered using the filter_alignment.py script^48^.

### Data availability

The raw sequence files supporting the findings of this article are available in the NCBI Sequence Read Archive (SRA) under the BioProject ID PRJNA356372, BioSamples SAMN06131047 to SAMN06131374.

### Statistical analysis

We used R software version 3.1.1^49^ together with RStudio software version 0.98.50751 for all statistical analyses as well as graphical representations of the data. We determined the reproducibility of our experiment using nucleic acid extracts from 17 faecal specimens randomly selected for processing in duplicate. Proportions of each OTU from each of the 17 faecal specimens were compared to the proportions from their technical repeats, using simple linear regression analysis to calculate the coefficient of determination (R^2^)^50^. We colour coded the plotted proportion of variations based on template nucleic acid concentrations, as well as sequencing depth.

We determined alpha and beta diversity using the Shannon diversity (H′)^51,52^ and the Bray Curtis dissimilarity index (calculated using the [vegdist] function from the R package vegan^53^)^52,54–56^, respectively. We performed agglomerative clustering by applying Complete Linkage (furthest neighbour) clustering using the [hclust] function in the R package stats^49^ together with a matrix based on the Bray-Curtis dissimilarity index. Clustering was performed on all OTUs with a relative abundance >0.5%. Due to the variation in the total number of reads sequenced across different specimens within a single run, we transformed count data^57^ to compositional data by calculating the relative abundance of each OTU per specimen^58,59^. As we were dealing with compositional data, we constructed log-ratio biplots^60^ using only OTUs where proportions differed significantly between participant groups (mothers, infants at birth, infants at 4–12 weeks and infants at 20–28 weeks) (at the 5% significance level). The data was adjusted in a Bayesian context to remove zeros^61–63^. Lambda scaling^64^ was employed to construct log-ratio biplots ensuring evenness in the spread of the modes (OTUs and specimens).

We used Fisher’s exact test for two-way tables or Pearson’s chi-square test to determine whether significant associations were present between covariates. We used generalized linear models (GLMs) to test the effect of covariates on the composition and diversity of maternal and infant faecal microbiota profiles, respectively. GLMs were used to test the effect of covariates at each of the time-points under study. We performed hypothesis testing at a 5% significance level, controlling for the false discovery rate as described by Benjamini & Hochberg^65^. We implemented the negative binomial model in RStudio^66^ through the family function quasipoisson; specified the offset as equal to “root OTU counts”^67^; and used paramaters estimated by the iterative weighted least squares method in the function [glm] in the package stats^49^. Final models were based on OTUs with a relative abundance >0.5%. These models were designed to test covariates with missing variables separately. Covariates included mode of delivery (vaginal delivery versus Caesarean-section delivery); gestational age; birth weight (low birth weight: <33 weeks gestation), birth length; gender; mode of feeding (exclusive breastfeeding, exclusive formula feeding or mixed feeding – a combination of breastfeeding and/or formula feeding and/or solid food); residential area [TC Newman (primarily a mixed ancestry population) vs. Mbekweni (primarily a black African population)]; maternal education; maternal HIV status; maternal smoking status; maternal cotinine levels; maternal body mass index (BMI) at 6–10 weeks postpartum; and the number of household members. Each statistically significant result obtained from our GLMs was graphically investigated to eliminate spurious results. In the event that significant variables showed uneven data distributions, we used “refitted GLMs” to validate our findings. Results from GLMs on uneven data could only be reported once confirmed by refitted GLMs. Refitted GLMs were based on subsets of comparable data points. To confirm the effect of maternal HIV status on faecal bacterial profiles, we used refitted GLMs to eliminate possible confounding effects of residential area and feeding practices. Therefore, refitted GLMs were based on data from participants residing in Mbekweni who did not practice exclusive formula feeding. The effect of mode of feeding on faecal bacterial profiles was confirmed by eliminating potential confounding effects of HIV status and residential area in the refitted GLMs. Here we only included data from HIV-infected mothers or HIV-exposed infants from Mbekweni. Refitted GLMs confirming the effect of residential area on faecal bacterial profiles included data from only HIV-uninfected mothers or HIV-unexposed infants which exclusively breastfed. Violin plots were used to summarise significant findings from our GLMs on all variables under study, including variables validated using refitted GLMs.

Differences in faecal bacterial profiles between mother-infant pairs, and amongst infants over time (using a subset of 36 infants with complete longitudinal data sets), were determined by generalized linear mixed models (GLMMs)^68^ using the glmmPQL function in the R package MASS^69^. In these models the same covariates (as above) were tested using GLMMs. Furthermore, these models also investigated possible interaction effects with each of the covariates and time (interaction-with-time models). A significant interaction effect would mean that the effect of the covariate differed across time-points. Significant findings from GLMs and GLMMs were also represented graphically; results were only deemed significant if the p-value and visual inspection of the related plots showed noticeable effects.

## Results

### Participant characteristics

Table 1 describes the characteristics of our study participants. We investigated the meconium bacterial profiles from 107 infants, of which the mothers of 90 infants provided a maternal faecal specimen at the time of delivery. Of the 107 infants investigated in this study, a subset of 72 and 36 infants had faecal specimens collected at 4–12 weeks and 20–28 weeks, respectively (Supplementary Fig. S1). The median age at which meconium samples were collected was 12 hours (Interquartile range (IQR): 4.2–24.6) postnatally, while infant faecal samples were collected at median ages of seven and 24 weeks during the two longitudinal collection periods (Table 1). The median age of maternal participants was 25 years (Table 1). Maternal faecal specimens collected from mothers undergoing vaginal delivery was 0 days (IQR: 0–1). Collections from mothers undergoing Caesarean-section deliveries took place at a median of two days after delivery (IQR: 1.5–2.5). Infants were primarily delivered vaginally (82%); had a median gestational age of 39 weeks; and a median birth weight of 3 kg (Table 1). We observed that some of our data were unevenly distributed across the groups under study (Supplementary Tables S4). Twenty percent of mothers were HIV-infected (18/90) (Table 1), of which the majority resided in Mbekweni (94%, 17/18) (Supplementary Table S1). Twenty four percent of the infants studied at birth were HIV-exposed (Table 1), but all were uninfected. Sixty nine percent of the HIV-exposed uninfected infants had a paired maternal sample. Most (85%) infants were exclusively breastfed prior to hospital discharge, while 47% and 17% were exclusively breastfed until 4–12 and 20–28 weeks, respectively (Table 1). All HIV-uninfected mothers exclusively breastfed prior to discharge, compared to 50% (9/18) of HIV-infected mothers (Supplementary Table S1). More HIV-unexposed infants were exclusively breastfed compared to HIV-exposed infants at birth (99%, 76/77 vs. 42%, 11/26), at 4–12 weeks (49%, 26/53 vs. 37%, 7/19), and at 20–28 weeks (17%, 6/36 vs. 0%, 0/10) (Supplementary Table S1). In addition, fewer HIV-unexposed infants were exclusively formula fed compared to those who were HIV-exposed at birth (1%, 1/77 vs. 58%, 15/26), at 4–12 weeks (2%, 1/53 vs. 53%, 10/19), and at 20–28 weeks (0%, 0/26 vs 70%, 7/10) (Supplementary Table S1). A subset of infants also received mixed feeding (a combination of breastfeeding and/or formula feeding and/or solid food introduction) at 4–12 weeks of age and 20–28 weeks (Table 1). Most mothers achieved a secondary-level of education and many were exposed to cigarette smoke (Table 1).

**Table 1:** Demographic and clinical characteristics of mothers and infants.

Characteristics	Mothers at delivery (n = 90)	Infants at birth (n = 107)	Infants at 4–12 weeks (n = 72)	Infants at 20–28 weeks (n = 36)
	Median (IQR) or N (%)	Median (IQR) or N (%)	Median (IQR) or N (%)	Median (IQR) or N (%)
Age at which specimens were collected	25.3 (22.2–30.1) years	12.4 (4.2–24.6) hours	7 (6.5–8.5) weeks	24 (23–26) weeks
Residential area:				
TC Newman (mixed ancestry population)	49 (54.4%)	49 (45.8%)	30 (41.7%)	16 (44.4%)
Mbekweni (black African population)	41 (45.6%)	58 (54.2%)	42 (58.3%)	20 (55.6%)
Maternal education:				
Primary level	8 (8.9%)	11 (10.3%)	8 (11.1%)	7 (19.4%)
Secondary level	76 (84.4%)	88 (82.2%)	60 (83.3%)	26 (72.2%)
Tertiary level	6 (6.7%)	8 (7.5%)	4 (5.6%)	3 (8.3%)
Maternal HIV status:				
HIV-infected	18 (20%)	—	—	—
HIV-uninfected	72 (80%)	—	—	—
Infant HIV status:				
HIV-exposed, uninfected	—	26 (24.3%)	19 (26.4%)	10 (27.8%)
HIV-unexposed, uninfected	—	81 (75.7%)	53 (73.6%)	26 (72.2%)
Maternal BMI (6–10 weeks postpartum)	25.8 (23.2–29.4)*	25.1 (23.0–29.1)*	25.8 (23.0–29.7)*	25.1 (22.6–29.4)*
Maternal smoking status:				
Active smoker (cotinine levels ≥ 500)	29 (32.2%)	31 (29.0%)	23 (31.9%)	14 (38.9%)
Passive smoker (cotinine levels > 10 < 500)	39 (43.3%)	48 (44.9%)	30 (41.7%)	13 (36.1%)
Non-smoker (cotinine levels ≤ 10)	22 (24.4%)	28 (26.2%)	19 (26.4%)	9 (25%)
Mode of delivery:				
Vaginal delivery	76 (84.4%)	88 (82.2%)	60 (83.3%)	29 (80.6%)
Caesarean-section delivery	14 (15.6%)	19 (17.8%)	12 (16.7%)	7 (19.4%)
Gestational age (weeks)	39 (37.3–40)	39 (38–40)	39 (38–40)	39 (38.8–40)
Birth weight (kg)	3 (2.7–3.3)	3 (2.7–3.3)	3.1 (2.7–3.4)	3 (2.6–3.3)
Birth length (cm)	51 (48.3–52)	50 (48–53)	51 (49–53)	50.5 (49–53)
Gender:				
Male	—	48 (44.9%)	33 (45.8%)	18 (50%)
Feeding:				
Exclusive breastfeeding	77 (89.5%)^†⨕^	87 (84.5%)^†⨕^	33 (46.5%)^†,φ^	6 (16.7%)^†⤉^
Exclusive formula feeding	9 (10.5%)^†⨕^	16 (15.5%)^†⨕^	11 (15.5%)^†,φ^	7 (19.4%)^†⤉^
Mixed feeding (breast milk, formula milk and/or solids)	—	—	27 (38%)^†,φ^	23 (63.9%)^†⤉^
Household members	5 (3–6)	4 (3–6)	4 (3–6)	4 (3–6)

*Number of observations recorded: Mothers at delivery: 56; Infants at birth: 69; Infants at 4–12 weeks: 53; Infants at 20–28 weeks: 27.

^†^Number of observations recorded: Mothers at birth: 86; Infants at birth: 103; Infants at 4–12 weeks: 71; Infants at 20–28 weeks: 36.

^⨕^Feeding recorded prior to discharge from hospital.

^φ^Feeding recorded at 4–12 weeks.

^⤉^Feeding recorded at 20–28 weeks.

IQR: Interquartile range. HIV: Human immunodeficiency virus.

### Meconium bacterial profiles are distinct from maternal faecal bacterial profiles at the time of delivery

**Figure 1:**
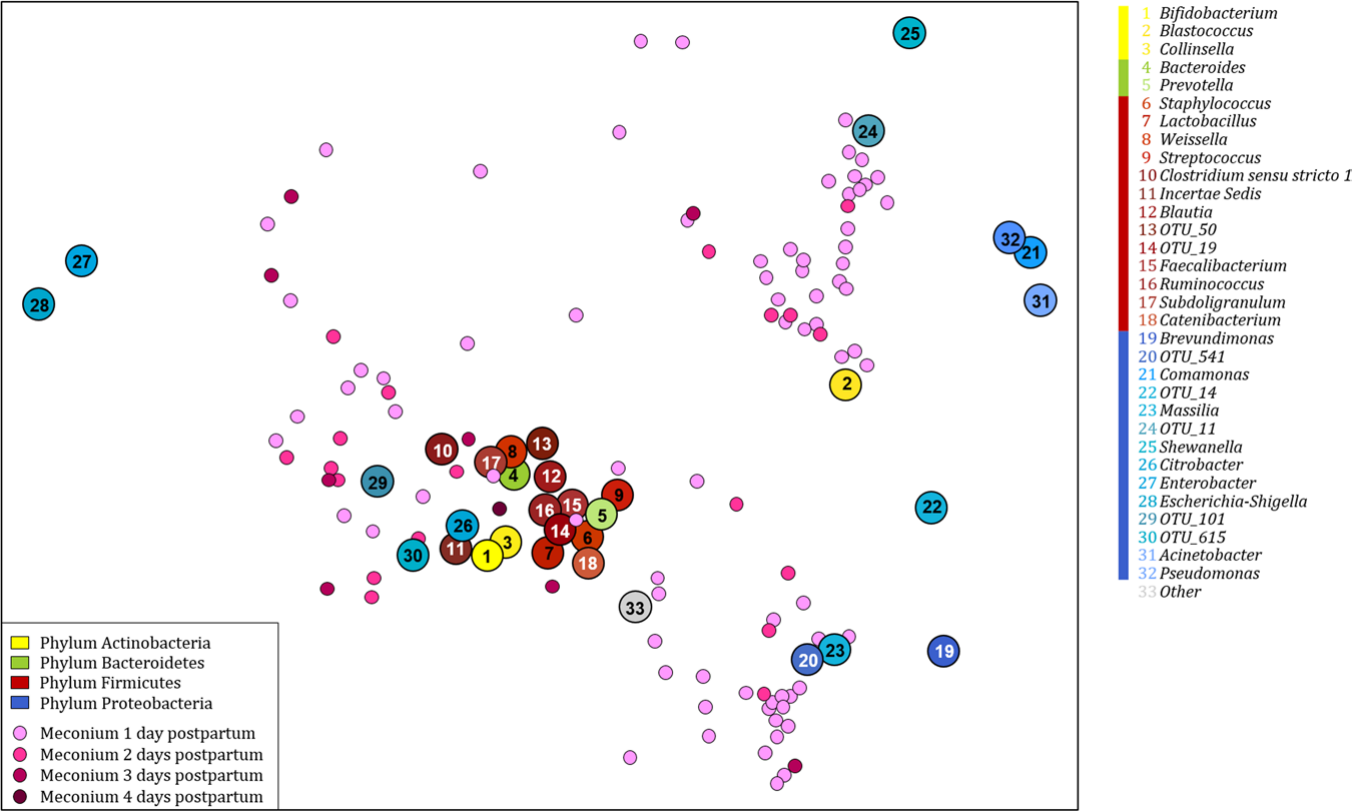
Log ratio biplot of infant meconium specimens, sampled during the first four days of life, in relation to the proportions of bacterial genera present in each of the specimens. Infant meconium specimens sampled during the first 24 hours of life are shown in light pink and specimens sampled between 24–48 hours, 48–72 hours and at more than 72 hours of life are shown in darker shades of pink. Genera present at proportions >0.5% in meconium specimens are colour-coded according to the phylum to which they belong (Yellow: Actinobacteria, Green: Bacteroidetes, Red: Firmicutes, Blue: Proteobacteria). The plot simultaneously represents the samples and genera taking the compositional nature of sequencing data into account. Connecting two genera and projecting the samples onto the connecting line indicates the log ratio of abundance of the genera in each sample. Unique operational taxonomic unit (OTU) numbers are assigned to unclassified taxa.

Clustering patterns of meconium specimens did not appear to be associated with timing of specimen collection at birth (Fig. 1). When compared to maternal faecal specimens, infant meconium had significantly lower alpha diversity indices [Shannon-index (H′ = 2.6 (IQR = 1.9–3.1) vs. (H′ = 2.9 (IQR = 2.7–3.1); (p < 0.001)]. Infant meconium and maternal faecal specimens also had distinct bacterial compositions at the time of delivery (Supplementary Table S2). Table 2 summarises significant differences in bacterial proportions at phylum- and class-level observed for 90 mothers and their infants studied at the time of delivery.

**Table 2:** Significant differences in bacterial proportions at phylum- and class-level observed for the 90 mother-infant pairs studied at the time of delivery.

	Infants at birth (n = 90) Median (IQR)	Mothers at birth (n = 90) Median (IQR)	p-value
Phylum Proteobacteria	**88.9** (**72.8–96.2)**	3.2 (1.2–8.9)	<0.001
Alphaproteobacteria	**7.6** (**0.5–24.3)**	0.1 (0.1–0.2)	<0.001
Betaproteobacteria	**15.9** (**0.4–25.4)**	0.1 (0.1–0.2)	<0.001
Gammaproteobacteria	**45.4** (**10.2–75.3)**	1.7 (0.8–7.4)	<0.001
Phylum Firmicutes	2.2 (0.9–16.8)	**66.4** (**57.3–77.2)**	<0.001
Bacilli	**1.2** (**0.5–4.6)**	1.0 (0.4–2.6)	0.0001
Clostridia	0.2 (0.1–1.7)	**49.2** (**38.8–61.3)**	<0.001
Erysipelotrichia	0.0 (0.0–0.1)	**11.1** (**7.1–13.9)**	<0.001
Phylum Actinobacteria	4.0 (1.7–10.4)	**14.5** (**6.4–24.5)**	<0.001
Actinobacteria	3.7 (1.6–10.2)	**11.2** (**3.3–21.6)**	<0.001
Coriobacteriia	0.0 (0.0–0.1)	**2.1** (**1.4–3.3)**	<0.001
Phylum Bacteroidetes	0.2 (0.0–0.7)	**5.0** (**1.7–12.3)**	<0.001
Bacteroidia	0.0 (0.0–0.1)	**5.0** (**1.7–12.3)**	<0.001

Bacterial proportions are rounded to one decimal point. Larger proportions of bacterial taxa are highlighted in bold.

IQR: Interquartile range.

**Figure 2:**
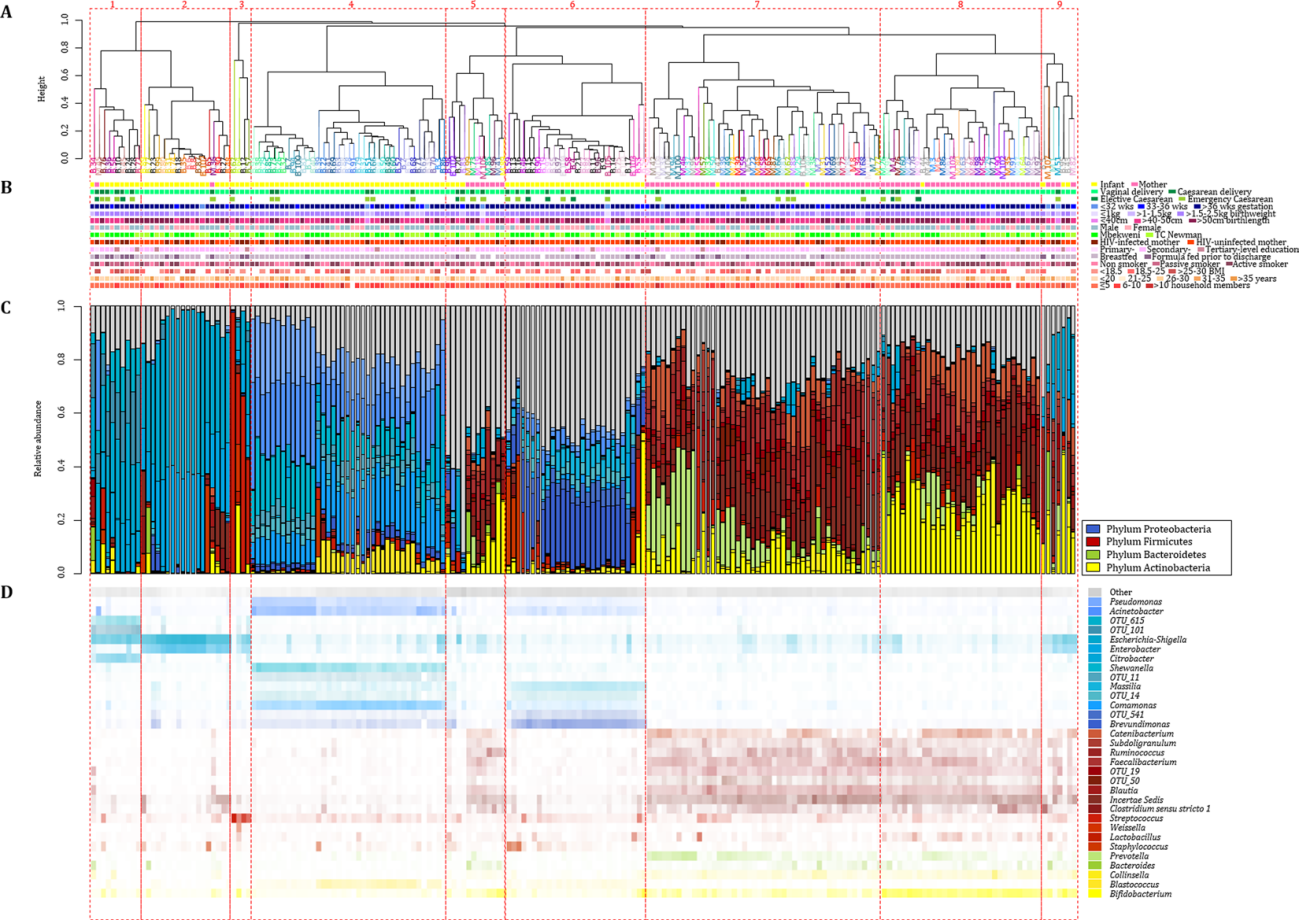
Infant and maternal faecal specimens form distinct clusters at the time of delivery, unsupervised clustering. (**A**) Dendogram representing 9 clusters for infant (n = 90) and maternal (n = 90) faecal specimens collected at birth. Infant faecal specimens are denoted by a “B” and maternal specimens by an “M”. Mother-infant pairs are denoted by a unique number and colour. (**B**) Participant demographics including sampling group, mode of delivery, infant gestational age (weeks), infant birth weight (kilograms) and infant birth length (centimetres), gender, population group, maternal HIV (Human immunodeficiency virus) status, maternal education, mode of feeding at birth, maternal smoking status, maternal body mass index (BMI) at 6–10 weeks postpartum, maternal age (years) and number of household members. (**C**) Relative abundance of faecal bacteria at genus-level. (**D**) Heatmap of the relative abundance of faecal bacteria at genus-level. Unique operational taxonomic unit (OTU) numbers are assigned to unclassified taxa.

Unsupervised clustering patterns at genus-level showed that maternal faecal specimens predominantly contained Firmicutes and clustered together (clusters 7 and 8) (Fig. 2). Meconium specimens had high abundances of Proteobacteria and formed clusters 1, 2, 3, 4 and 6 (Fig. 2). Clusters no. 5 and 9 had a mix of meconium and maternal faecal specimens, however, no mother-infant pairs grouped within these clusters (Fig. 2). Overall, only two mother-infant pairs grouped closely together in clusters no. 8 (mother-infant pair no. 44) and no. 1 (mother-infant pair no. 45) (Fig. 2A). Apart from the distinction between maternal and infant faecal bacterial profiles at the time of delivery, no other characteristics were associated with these profiles using unsupervised clustering (Fig. 2B). Similar clustering profiles were observed when each genus, irrespective of its relative abundance, was treated as a single unit during unsupervised clustering (data not shown).

### Changes in infant faecal bacterial profiles over the first 28 weeks of life and their correlation with maternal faecal specimens collected at birth

**Figure 3:**
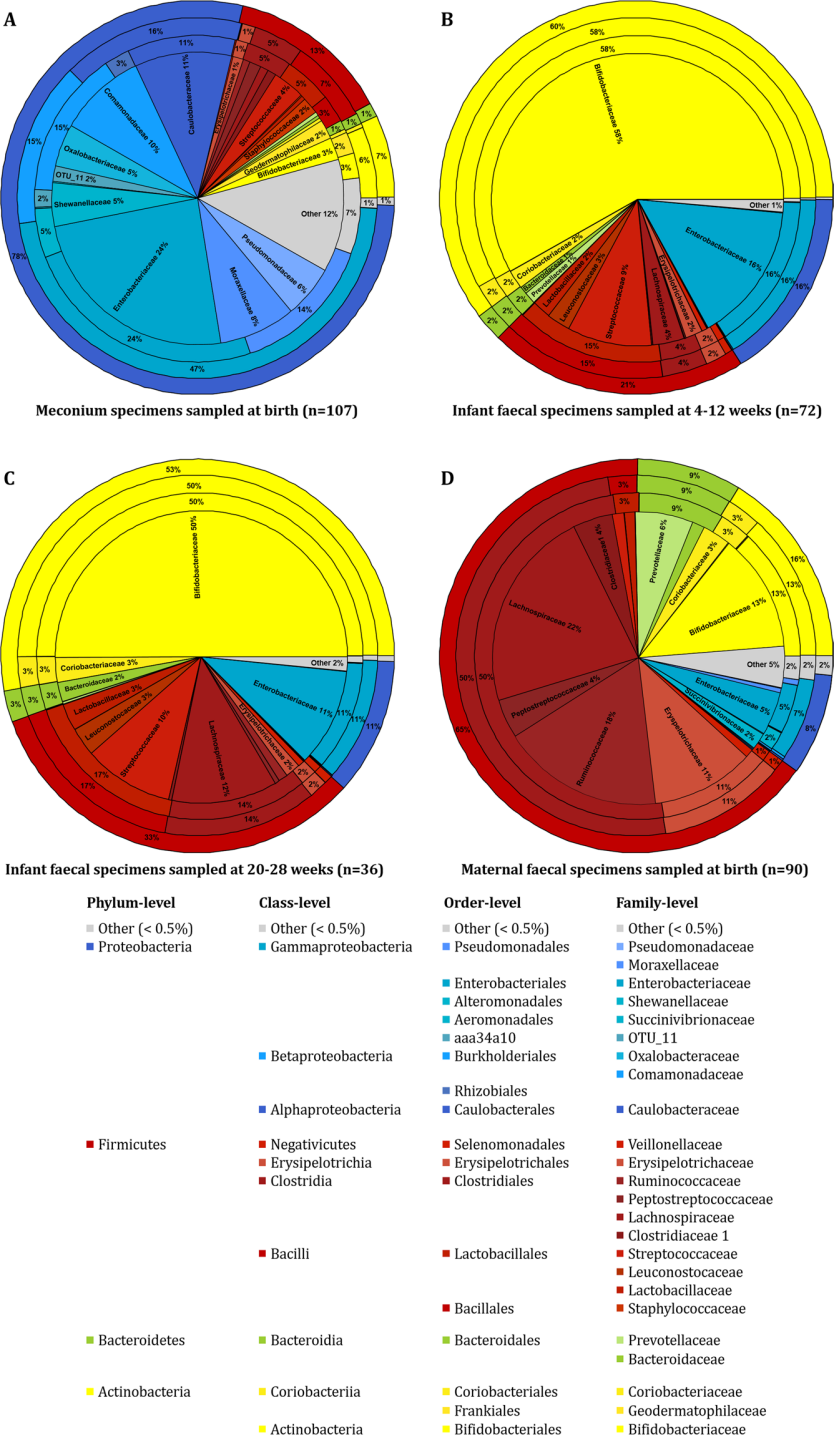
Bacterial composition of all infant and maternal faecal specimens under study. The outer ring of each pie chart represents the bacterial composition at phylum-level; the second ring from the outside represents the bacterial composition at class-level; the third ring represents the order-level and central ring of each pie chart represents the bacterial composition at family-level. (**A**) Infant meconium specimens (n = 107); (**B**) infant faecal specimens sampled at 4–12 weeks of age (n = 72); (**C**) infant faecal specimens sampled at 20-28 weeks of age (n = 36); and D) maternal faecal specimens sampled at delivery (n = 90). Unique operational taxonomic unit (OTU) numbers are assigned to unclassified taxa.

Infant meconium samples had higher alpha diversity measures [H′ = 2.6 (IQR = 1.9–3.1)] than infant faecal specimens collected at 4–12 weeks [H′ = 1.1 (IQR = 0.7–1.7)] and 20–28 weeks of age [H′ = 1.5 (IQR = 1.2–1.9)] (p < 0.001). Figure 3 shows the temporal evolution of faecal bacterial profiles of all infants under study during the first 28 weeks of life (Supplementary Table S3). Meconium bacterial profiles from 107 infants at birth were distinct from bacterial profiles of faecal specimens collected from the subset of infants at 4–12 weeks (n = 72) and 20–28 (n = 36) weeks of life (Fig. 3A–C). Maternal faecal specimens (n = 90) had a distinctly different bacterial composition when compared to infant meconium and faecal specimens (Fig. 3A–D).

**Figure 4:**
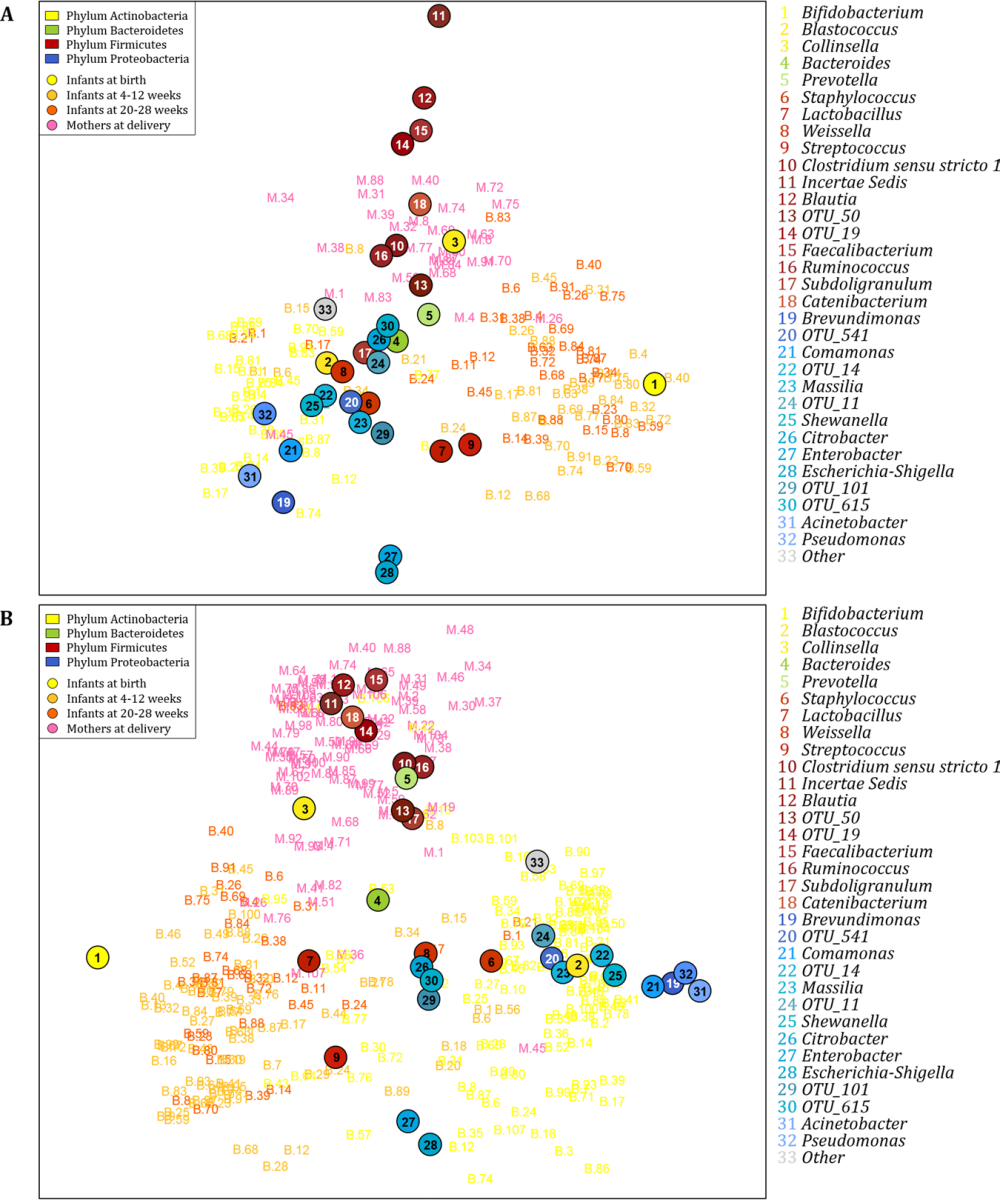
Log ratio biplot of mother-infant pairs at the time of delivery, 4–12 weeks and 20–28 weeks of life in relation to the proportions of bacterial genera present in each of the specimen (**A**) Log ratio biplot of 36 mothers and their infants with complete longitudinal sets. (**B**) Log ratio biplot of 90 mothers, 107 infants sampled at the time of delivery, 72 infants sampled at 4–12 weeks, and 36 infants sampled at 20–28 weeks. Infant meconium specimens are shown yellow, infant faecal specimens sampled at 4–12 weeks of age are shown in light orange, infant faecal specimens sampled at 20–28 weeks of age are shown in dark orange and maternal faecal specimens are shown in pink. Genera present in faecal specimens with proportions >0.5% are colour-coded according to the phylum to which they belong (Yellow: Actinobacteria, Green: Bacteroidetes, Red: Firmicutes, Blue: Proteobacteria). The plot simultaneously represents the samples and genera taking the compositional nature of sequencing data into account. Connecting two genera and projecting the samples onto the connecting line indicates the log ratio of abundance of the genera in each sample. Unique operational taxonomic unit (OTU) numbers are assigned to unclassified taxa.

Log ratio biplot analysis (at genus-level) on the 36 infants with complete longitudinal sets (Fig. 4A), revealed similar clustering profiles as seen for the complete data set (Fig. 4B). Of note, we observed that infant faecal specimens sampled at 4–12 and 20–28 weeks were dominated by *Lactobacillus*, *Streptococcus* and *Bifidobacterium* and overlapped (Fig. 4A and B). Maternal faecal and infant meconium specimens collected at the time of delivery were distinct from each other and from infant faecal profiles at 4–12 and 20–28 weeks (Fig. 4A and B). Maternal faecal specimens primarily clustered around genera representing the class Clostridia. Infant meconium specimens clustered around Alpha-, Beta- and Gammaproteobacteria. We also investigated whether faecal bacterial profiles from the three infant age groups (birth, 4–12 weeks and 20–28 weeks of life), where complete longitudinal sample sets were available (n = 36), were significantly different (Supplementary Table S4; Supplementary Fig. S2).

### The effect of maternal HIV status, mode of feeding and residential area on faecal bacterial profiles

**Figure 5:**
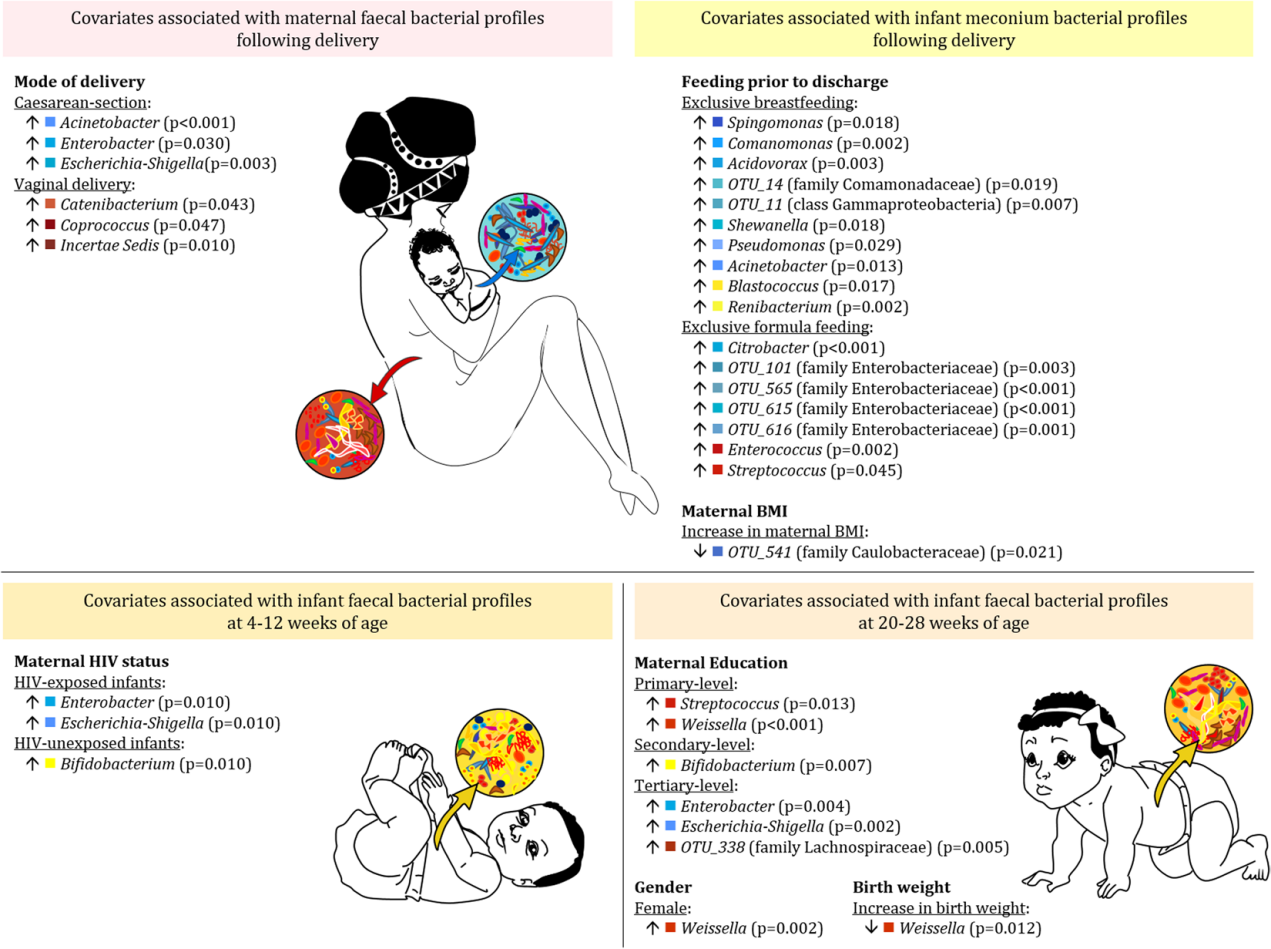
Covariates significantly associated with faecal bacterial genera from infant and maternal faecal specimens. Bacterial genera significantly associated with covariates assessed in this study are summarized. When comparing proportions of bacterial genera observed among categorical covariates, higher proportions are denoted by up arrows. Up or down arrows next to numerical covariates indicate significant associations observed with either an increase or decrease in the proportion of significant genera associated with these numerical covariates. Unique operational taxonomic unit (OTU) numbers are assigned to unclassified taxa.

GLMs incorporating all covariates and full datasets from the groups under study showed that infant meconium bacterial profiles were influenced by mode of feeding (Supplementary Fig. S3). Meconium from infants exclusively breastfed prior to discharge (87/103) had significantly higher proportions of bacteria within the phyla Proteobacteria and Actinobacteria when compared to those exclusively formula fed (Supplementary Fig. S3; Fig. 5). Infants exclusively formula fed prior to discharge had significantly higher proportions of the family Enterobacteriaceae (p = 0.039), including the genera *Enterococcus* (p = 0.002) and *Streptococcus* (p = 0.045) (Fig. 5). GLMs showed that maternal faecal bacterial profiles were significantly associated with maternal HIV status, mode of feeding as well as residential area (Table 3). Faecal bacterial profiles of infants at 4–12 weeks were also significantly associated with maternal HIV status, feeding practices and residential area (Table 3). At 20–28 weeks, infant faecal bacterial profiles were associated with maternal HIV status and mode of feeding (Table 3).

**Table 3:** Variables with uneven data distribution significantly associated with maternal and/or infant faecal bacterial profiles.

	Significant covariates using GLMs			Significant covariates tested using “refitted GLMs”		
	Maternal HIV status	Feeding practices prior to discharge	Residential area	Maternal HIV status	Feeding practices prior to discharge	Residential area
**Mothers (n = 90)**						
Phylum Proteobacteria	0.045*	0.020*	—	0.049*	0.029*	—
Class Gammaproteobacteria	0.014*	—	—	0.031*	0.026*	—
Order Enterobacteriales	<0.001*	—	—	0.009*	0.034*	—
Family Enterobacteriaceae	0.027*	—	0.002*	0.009*	0.034*	—
*Escherichia*-*Shigella*	—	—	0.040*	—	—	—
Class Deltaproteobacteria	0.030*	—	—	—	0.043*	—
Order Desulfovibrionales	0.040*	—	—	—	0.043*	—
Family Desulfovibrionaceae	0.016*	—	—	—	0.043*	—
Class Bacilli	0.004*	—	—	0.002*	—	—
Order Lactobacillales	0.003*	—	—	0.003*	—	—
Family Streptococcaceae	0.040*	—	0.004*	0.024*	—	—
*Streptococcus*	—	—	0.012*	—	—	—
Family Ruminococcaceae	0.030*	—	—	—	—	—
Phylum Actinobacteria	—	0.020*	—	—	0.017*	—
Class Actinobacteria	—	0.030*	—	—	0.012*	—
Order Bifidobacteriales	—	0.020*	—	—	0.012*	—
Class Coriobacteriia	0.030*	—	—	0.013*	—	—
Order Coriobacteriales	0.038*	—	—	0.013*	—	—
Family Coriobacteriaceae	0.027*	—	—	0.013*	—	—
Class Bacteroidia	0.043*	—	—	—	—	—
**Infants at 4–12 weeks**						
Phylum Proteobacteria	0.005*	—	—	0.043*	—	—
Class Gammaproteobacteria	0.006*	—	—	0.041*	—	—
Order Enterobacteriales	0.006*	—	—	0.041*	—	—
Family Enterobacteriaceae	0.007*	—	—	0.041*	—	—
*Enterobacter*	0.010*	—	—	0.036*	—	—
*Escherichia*-*Shigella*	0.010*	—	—	0.039*	—	—
Phylum Firmicutes	—	0.011*	0.002*	—	—	—
Class Bacilli	—	0.020*	0.036*	—	—	—
Order Lactobacillales	—	0.020*	0.036*	—	—	—
Family Leuconostocaceae	—	0.001*	—	—	—	—
*Weissella*	—	0.001*	—	—	—	—
Class Erysipelotrichia	—	—	0.040*	—	—	—
Order Erysipelotrichiales	—	—	0.040*	—	—	—
Class Negativicutes	0.006*	—	0.036*	—	—	—
Order Selenomonadales	0.006*	—	0.036*	—	—	—
Family Veillonellaceae	0.005*	—	0.034*	—	—	—
Phylum Actinobacteria	0.005*	<0.001*	—	0.047*	—	—
Class Actinobacteria	0.006*	<0.001*	—	0.045*	—	—
Order Bifidobacteriales	0.006*	<0.001*	—	0.045*	—	—
Family Bifidobacteriaceae	0.007*	<0.001*	—	0.045*	—	—
*Bifidobacterium*	0.010*		—	0.045*	—	—
Class Coriobacteriia	—	0.020*	—	—	—	—
Order Coriobacteriales	—	0.020*	—	—	—	—
Family Coriobacteriaceae	—	0.024*	—	—	—	—
*Collinsella*	—	0.035*	—	—	—	—
**Infants at 20–28 weeks**						
Class Erysipelotrichia	0.030*	—	—	—	—	—
Order Erysipelotrichiales	0.030*	—	—	—	—	—
Phylum Actinobacteria	0.040*	0.001*	—	—	—	0.040*
Phylum Bacteroides	0.030*	—	—	<0.001*	—	—
Class Bacteroidia	0.030*	—	—	<0.001*	—	—
Order Bacteroidales	0.030*	—	—	<0.001*	—	—

^*^Significant p-values; Non-significant p-values are shown by “−”.

*P-values in bold highlights true significant results from generalised linear models (GLM)s as validated by “refitted GLMs”*. HIV: Human immunodeficiency virus.

A detailed exploratory investigation of the variables maternal HIV status, mode of feeding and residential area showed an uneven distribution of data across the participants under study (Supplementary Table S1). Based on our refitted models, used to validate the effect of these three variables, we found that only maternal HIV status and mode of feeding had true significant effects on maternal faecal bacterial profiles (Table 3). Infant faecal bacterial profiles at 4–12 and 20–28 weeks were only influenced by maternal HIV status (Table 3).

Maternal faecal bacterial profiles were influenced by both their HIV status and feeding practices (Table 3; Supplementary Figs S4 and S5). Mothers who exclusively breastfed prior to discharge (77/86) also had higher faecal diversity compared to mothers who exclusively formula fed [H′ = 2.9 (IQR: 2.7–3.1) vs. H′ = 2.8 (IQR: 2.6–2.8); p = 0.034]. Infant faecal bacterial profiles at 4–12 weeks were significantly associated with maternal HIV status (Table 3; Supplementary Fig. S6). We found that HIV-exposed infants (19/72) had higher faecal bacterial diversity compared to HIV-unexposed infants (53/72) [H′ = 1.8 (IQR: 1.1–1.9) vs. H′ = 1.0 (IQR: 0.6–1.3); p = 0.026]. Feeding practice also significantly impacted on faecal bacterial diversities at 4–12 weeks of age. Infants exclusively formula fed (11/71) up to the time of sample collection had highest faecal bacterial diversity followed by infants receiving mixed feeding (27/71) and exclusive breastfeeding (33/71) over this period (H′ = 1.9 (IQR: 1.7–2.0) vs. H′ = 1.2 (IQR: 0.7–1.5) vs. H′ = 1.0 (IQR: 0.6–1.3); p = 0.024]. At 20–28 weeks, HIV-exposed infants (10/36) had significantly higher proportions of the order Bacteroidales (p = 0.030) when compared to HIV-unexposed infants (Table 3; Supplementary Fig. S7). Infants at 20–28 weeks also had higher faecal bacterial diversity [H′ = 2.0 (IQR: 1.7–2.4) vs. H′ = 1.3 (IQR: 0.9–1.7); p = 0.002].

The effect of HIV-exposure on infant faecal bacterial profiles over time was assessed using an interaction-with-time model. This model included complete datasets from the 36 infants with samples collected from birth until 20–28 weeks. We found that Proteobacteria proportions decreased with an increase in age among HIV-unexposed infants (Supplementary Fig. S8A). In contrast, Clostridia proportions increased with an increase in age among HIV-unexposed infants (Supplementary Fig. S8A). A less prominent effect of infant age on faecal bacterial profiles was observed among HIV-exposed infants (Supplementary Fig. S8B). Our interaction-with-time model also showed that infants exclusively formula fed had the highest faecal bacterial diversity measured during the first 28 weeks of life [birth: H′ = 1.8 (IQR: 1.7–2.4); 4–12 weeks: H′ = 1.9 (IQR: 1.7–2.0); 20–28 weeks: H′ = 2.3 (IQR: 2.0–2.5)]. Diversity indices increased with an increase in infant age (p = 0.006). Infants who were mixed fed also showed a slight increase in diversity over time [4–12 weeks: H′ = 1.2 (IQR: 0.7–1.5); 20–28 weeks: H′ = 1.3 (IQR: 1.1–1.7)]. Infants exclusively breastfed throughout the first 28 weeks of life showed a decrease in infant faecal bacterial diversity as their age increased [birth: H′ = 2.7 (IQR: 2.1–3.1); 4–12 weeks: H′ = 1.0 (IQR: 0.6–1.3); 20–28 weeks: H′ = 0.9 (IQR: 0.9–1.9)].

### Mode of delivery and its influence on infant and maternal faecal bacterial profiles

Figure 5 summarises significant associations between mode of delivery and maternal faecal bacterial profiles at genus-level. Mothers delivering via C-section delivery (14/90) had significantly higher proportions of *Escherichia-Shigella* (p = 0.003), *Acinetobacter* (p < 0.001) and *Enterobacter* (p = 0.03) when compared to mothers undergoing vaginal delivery (Supplementary Fig. S9). Higher proportions of *Catenibacterium* (p = 0.04), *Coprococcus* (p = 0.047), and *Incertae Sedis* (p = 0.01) were observed from mothers delivering via vaginal delivery. Fischer exact tests for two way tables showed that infant birth weight (p = 0.018), birth length (p = 0.008), as well as the period between delivery and sample collection (p < 0.001) were significantly associated with mode of delivery; however, none showed any direct relationship with maternal faecal bacterial profiles. We further tested whether other potential confounders, such as anaemia, hypertension and urinary tract infections recorded during pregnancy; anaesthetic use during labour; analgesic use during labour; oxytocin-like substance use during labour; the period between membrane rupture and delivery; as well as any medication supplementation during hospitalisation, were significantly associated with delivery mode. Of these, only anaesthetic use during labour was significantly associated with mode of delivery (p < 0.001).

### Maternal body mass index and education, infant gender, birth weight and gestational age also influences maternal and/or infant faecal bacterial profiles

High maternal BMI (measured at 6–10 weeks postpartum) was associated with lower proportions of Alphaproteobacteria (RR = 0.80; p = 0.030) and Bacilli (RR = 0.70; p = 0.030) from meconium, whilst Gammaproteobacteria were positively correlated with higher maternal BMI (RR = 1.09; P = 0.024). High maternal BMI was also associated with increased proportions of Veillonellaceae (RR = 1.46; p = 0.025) at 20–28 weeks of age. No correlations were observed when plotting maternal BMI and Firmicutes/Bacteroidetes proportions against maternal BMI (Supplementary Fig. S10). Maternal education was associated with significant differences in the proportions of Gammaproteobacteria, Clostridia and Actinobacteria observed from maternal faecal specimens (Supplementary Fig. S11A). Maternal education also had a significant association with infant faecal bacterial profiles measured at 20–28 weeks of life (Supplementary Fig. S11B). Infant gender and birth weight was associated with faecal bacterial composition only at 20–28 weeks of life. Female infants had significantly higher proportions of the family Leuconostocaceae (p = 0.001) and the genus *Weissella* (p = 0.002). Infant birth weight was inversely associated with proportions of the families Leuconostocaceae (RR = 0.03; p < 0.001) and Ruminococcaceae (RR = 0.003; p < 0.001) as well as the genus *Weissella* (RR = 0.03; p = 0.012).

## Discussion

This study is the first to investigate infant meconium bacterial profiles in an African setting. Infant meconium specimens contain high abundances of the phylum Proteobacteria^70,71^. High proportions of the phylum Proteobacteria from meconium specimens in our study is in agreement with the finding by Ardissone and colleagues^2^ reporting high abundances of Proteobacteria from meconium of infants born at a gestational age of more than 33 weeks. Another interesting finding was the positive correlation between maternal BMI measures and the class Gammaproteobacteria from meconium. No differences have previously been shown between the meconium bacterial composition of infants born to overweight or obese (OWOB) mothers and those born to mothers with normal BMIs^72^. In contrast, differences have been reported when assessing faecal bacterial profiles collected from older infants^72^.

Infant faecal bacterial profiles have also been shown to be influenced by early life feeding practices^17–19^. In our study, the effect of mode of feeding was already evident from meconium specimens. Meconium from infants exclusively formula fed had significantly higher proportions of the family Enterobacteriaceae compared to those exclusively breastfed. Feeding has also been reported to promote shifts from the highly variable infant-like GIT bacterial profile towards a more stable adult-like profile when solid foods are introduced^73^. Primary shifts in infant faecal bacterial profiles during the introduction of solid foods are characterized by a significant reduction in Bifidobacteria^20^; an increase in Clostridia^20^ and Bacteroidetes^74,75^; a reduction in the proportion of facultative anaerobes; and an increase in the overall microbial diversity^20^. Although we did not test the effect of solid food on infant faecal bacterial profiles, we did observe a slight increase in Bacteroidia and Clostridia amongst infants at 20–28 weeks of age.

In addition, we showed the importance of exploratory analysis in observational studies such as ours. Observational studies, as opposed to designed experiments, potentially allows for the investigation of uneven datasets and reports of spurious p-values as a result thereof. In this study, each statistically significant p-value was carefully investigated graphically in order to assess whether further validation of the results were needed. For example, one of our observations was that HIV-infected mothers primarily opted for exclusive breast- or mixed feeding practices whilst HIV-infected mothers tended to practice formula feeding. In our initial analysis, we found that both HIV-unexposed as well as breastfed infants had higher proportions of beneficial *Bifidobacterium*^76–78^. Upon validation of our results, we concluded that maternal HIV status was the primary driver of this observation among infants in our cohort. Of note, in South Africa, HIV-infected mothers tend to formula feed^31^ despite recommendations from the World Health Organization^32^. This is relevant, given the important health benefits that breast milk provide, including for HIV-exposed children^29,30^.

In this study, we also found low proportions of Bacteroidetes among our adult population contrary to previous reports^79^. Reduced proportions of Bacteroidetes in our cohort compared to other adult participants could be ascribed to the fact that we only studied faecal bacterial profiles from females. Two previous studies, one from the United States of America and one from four European locations (France, Germany, Italy, and Sweden), reported lower abundances of Bacteroidetes among adult females compared to males^80,81^. An alternative potential explanation for the low Bacteroidetes proportions observed from our mothers may be their BMI, since the majority of moms participating in our study was overweight or obese. Overweight/obesity has been previously associated with reduced levels of Bacteroidetes and increased levels of Firmicutes^82–84^. The latter, however, was not observed in our study (Supplementary Fig. S10). In addition to the low proportions of Bacteroidetes observed from mothers under study, we noted that mode of delivery influenced maternal faecal bacterial profiles. The mechanism by which this occurs is unknown. We hypothesize that stress may be a potential contributor to changes in maternal faecal bacterial profiles during delivery. Studies have shown that stress may alter the maternal GIT^85^ and vaginal microbiota^85,86^. In addition, it has been reported that maternal prenatal stress may contribute to shifts in infant faecal bacterial profiles^87^. We also thought of investigating the effect of the process of delivery, in particular anaesthetic use during delivery, on maternal faecal bacterial profiles. Although we found a significant association between mode of delivery and anaesthetic use, we could not establish whether the use of anaesthetics or some other aspect of Caesarean-section delivery caused these changes. This was primarily due to the fact that anaesthetics were given to all mothers undergoing Caesarean-section delivery. In support of the hypothesis that mode of delivery may modulate maternal faecal microbiota profiles, previous reports have shown some influence on maternal colostrum and breastmilk as well as vaginal bacterial profiles^88–91^. We further found that feeding practices had a significant effect on maternal faecal bacterial profiles. This is surprising given the short time-frame between the birth process and specimen collection from mothers. The bacterial entero-mammary pathway^92^ could potentially support this finding due to the potential crosstalk between the maternal GIT and mammary glands. Nevertheless, further investigation is needed to support our finding.

## Limitations

In this study, one of the main limitations was the drop in the number of infants assessed at follow-up time-points. Among the 107 infants studied at birth, only 36 were followed longitudinally up until 20–28 weeks. Missed samples were primarily due to the inability of infants to pass stool at the scheduled study visits. In addition, some infants could not attend the scheduled stool sample collection visit. Hence, significant findings from the reduced sample size of 36 assessed at 20–28 weeks of life may need to be interpreted with caution due to the variable nature of the faecal microbiota composition. However consistencies of results across age groups in our analysis provide confidence that significant effects displayed by our data are indeed true effects. Another potential limitation of this study might be the sequencing depths obtained for faecal specimens under investigation as previously highlighted by Ni and colleagues^93^. However, we are confident that the sequencing depth obtained in our study was sufficient to answer our research questions (Supplementary Fig. S12)^93,94^. We did not observe any effect of sequencing depth on the clustering profiles observed for the different groups under study (Supplementary Fig. S13). In contrast, we noted that template concentration had a bigger effect on reproducibility compared to sequencing depth (Supplementary Fig. S14).

## Conclusion

The meconium from infants investigated in our study contained high proportions of the phylum Proteobacteria, in particular bacteria within the Enterobacteriaceae family. Infant meconium was distinct from maternal faecal bacterial profiles sampled at birth. In addition, infant faecal bacterial profiles changed during the first 28 weeks of life. Major determinants of infant meconium bacterial profiles were mode of feeding and maternal BMI. HIV-exposure, on the other hand, was an important contributor to the composition of infant faecal bacterial profiles at 4–12 weeks of life, with HIV-exposed infants having higher bacterial diversity and reduced proportions of Bifidobacteria. Maternal faecal bacterial profiles following delivery were also influenced by their HIV status, feeding practices, as well as mode of delivery. Further large longitudinal studies are needed to improve our understanding of the contribution of maternal HIV status and mode of delivery to infant and maternal faecal microbial profiles.

### Summary of 16S rRNA sequencing data output and reproducibility

Following the removal of potential contaminating reads we obtain a median of 5465 (IQR: 3159–9877) post-filtered reads per sample. Maternal faecal specimens sampled at birth had the lowest number of reads following correction for contamination (median: 3155; IQR: 2104–4355); followed by infants at 20–28 weeks (median: 5636; IQR: 4228–7084) and infants at 4–12 weeks (median: 6407; IQR: 4069–9042). Infants at birth had the highest number of reads sequenced from their meconium (median: 10002; IQR: 5065–14830). Rarefaction curve analysis indicates sufficient sequences for calculating Shannon diversity indices in our study (Supplementary Fig. S12).

According to Ni and colleagues, a decrease in sequencing depth results in an increase in dissimilarities (beta diversities) between microbial community samples^93^. Multidimensional scaling^95^ showed an effect of sequencing depth on dissimilarity indices (beta diversities)^54,55^ between samples in our study. Samples with low sequencing depths (in red) had higher beta diversities compared to samples with higher sequencing depths (in green and blue) (Supplementary Fig. S13A). Although we observed an effect of sequencing depth on beta diversities, this did not have a large enough influence to impact on beta diversities calculated between different sampling groups (Supplementary Fig. S13B). We observed distinct clusters between maternal faecal specimens; infant meconium specimens; and infant faecal specimens collected at 4–12 and 20–28 weeks of age, which corresponds to our log ratio biplot analysis (Fig. 4).

The subset of faecal specimens (n = 17) that were processed in duplicate confirms that our 16S rRNA sequencing technique was reproducible (adjusted R^2^ = 0.98). However, we determined that sequencing data was less reproducible where lower template concentrations used during library preparation (Supplementary Fig. S14A), as previously reported^96^. Of note, template concentration seemed to have a more pronounced effect on reproducibility compared to sequencing depth (Supplementary Fig. S14B). Mock communities (HM782-D and HM783-D) (BEI Resources, ATCC, VA, USA) were sequenced successfully with similar profiles generated as previously reported when targeting the V4 hypervariable region of the 16S rRNA gene^97^.

## Electronic supplementary material

Supplementary data
