# Supplementary material for: HIV-exposure, early life feeding practices and delivery mode impacts on faecal bacterial profiles in a South African birth cohort
Source: Sci Rep. 2018 Mar 22;8:5078. doi: 10.1038/s41598-018-22244-6 (PMC5864830; doi:10.1038/s41598-018-22244-6)
Supplement: Supplementary file 1 — Supplementary data [file 41598_2018_22244_MOESM1_ESM.pdf]

HIV-exposure, early life feeding practices and delivery mode impacts on faecal bacterial profiles in a South African birth cohort.

Authors' list:
Shantelle Claassen-Weitz[1], Sugnet Gardner-Lubbe[2], Paul Nicol[3], Gerrit Botha[3], Stephanie Mounaud[4], Jyoti Shankar[4], William C Nierman[4], Nicola Mulder[3], Shrish Budree[5,6], Heather J Zar[5,7,8], Mark P Nicol[1,8,9], Mamadou Kaba[1,8*]

Affiliations:
[1]Division of Medical Microbiology, Department of Pathology, Faculty of Health Sciences, University of Cape Town, Cape Town, South Africa;
[2]Department of Statistics and Actuarial Science, Faculty of Economic and Management Sciences, Stellenbosch University;
[3]Computational Biology Group and H3ABioNet, Department of Integrative Biomedical Sciences, University of Cape Town, Cape Town, South Africa;
[4]J. Craig Venter Institute, Rockville, Maryland, United States of America;
[5]Department of Paediatrics and Child Health, Red Cross War Memorial Children's Hospital, Cape Town, South Africa;
[6]OpenBiome, Somerville, Massachusetts, United States of America;
[7]SAMRC Unit on Child & Adolescent Health, University of Cape Town, Cape Town, South Africa;
[8]Institute of Infectious Disease and Molecular Medicine, Faculty of Health Sciences, University of Cape Town, Cape Town, South Africa
[9]National Health Laboratory Service of South Africa, Groote Schuur Hospital, Cape Town, South Africa.

Author e-mail addresses:

Shantelle Claassen-Weitz	:	tellafiela@gmail.com
Sugnet Gardner-Lubbe	:	slubbe@sun.ac.za
Paul Nicol	:	paul.nicol@classicsnetwork.com
Gerrit Botha	:	gerrit.botha@uct.ac.za
Stephanie Mounaud	:	sharris@jcvi.org
Jyoti Shankar	:	jyoti.shankar@gmail.com
William C Nierman	:	wnierman@jcvi.org
Nicola Mulder	:	nicola.mulder@uct.ac.za
Shrish Budree	:	BDRSHR001@myuct.ac.za
Heather J Zar	:	heather.zar@uct.ac.za
Mark P Nicol	:	mark.nicol@uct.ac.za
Mamadou Kaba	:	mamadou.kaba@hotmail.com

***Corresponding author:** Mamadou Kaba
Tel: +27 21 406 63 62
Fax: +27 21 406 6210
E-mail address: mamadou.kaba@hotmail.com

Postal address: University of Cape Town
Faculty of Health Sciences
Division of Medical Microbiology
Anzio Road, Observatory, 7925
Cape Town
South Africa

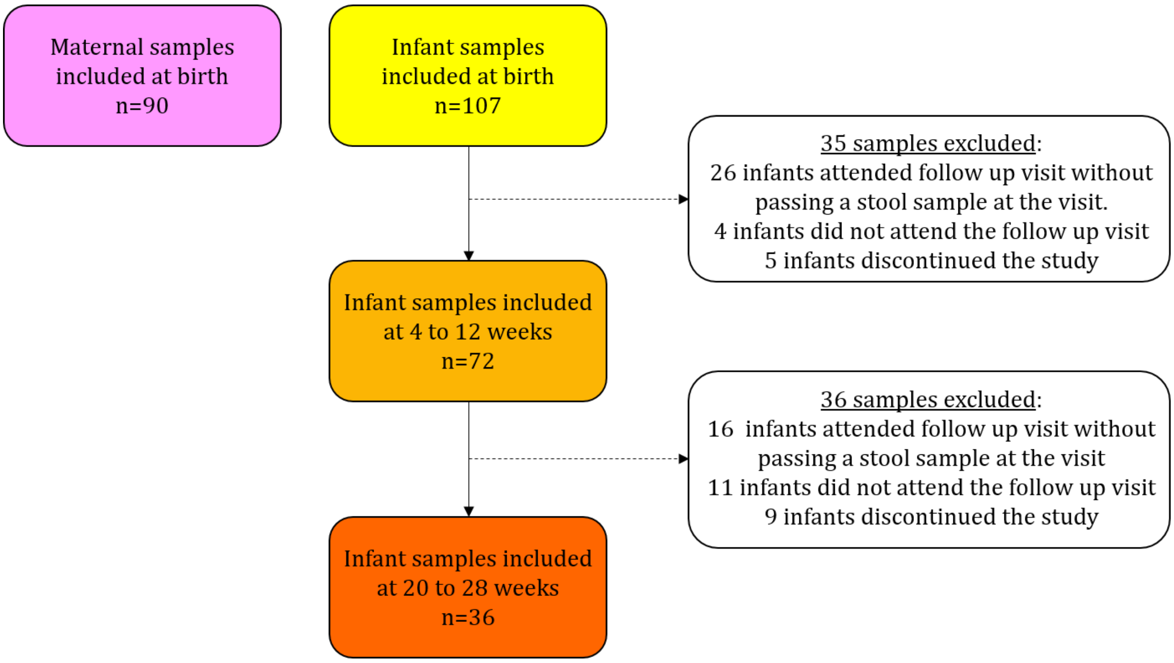

Supplementary Figure S1. Maternal and infant faecal specimens included in the study.

Supplementary Table S1. Distribution of maternal HIV status and feeding data across two population groups.

	Exclusively breastfed	Exclusively formula-fed	Mixed-fed	Missing data	Total
**Mothers at birth (n=90)**					
**Mbekweni**					
HIV-uninfected	23	0	0	1	**24**
HIV-infected	8	9	0	0	**17**
**Total**	**31**	**9**	**0**	**1**	**41**
**TC Newman**					
HIV-uninfected	45	0	0	3	**48**
HIV-infected	1	0	0	0	**1**
**Total**	**46**	**0**	**0**	**3**	**49**
**Infants at birth (n=107)**					
**Mbekweni**					
HIV-unexposed	31	1	0	1	**33**
HIV-exposed	10	15	0	0	**25**
**Total**	**41**	**16**	**0**	**1**	**58**
**TC Newman**					
HIV-unexposed	45	0	0	3	**48**
HIV-exposed	1	0	0	0	**1**
**Total**	**46**	**0**	**0**	**3**	**49**
**Infants at 4-12 weeks (n=72)**					
**Mbekweni**					
HIV-unexposed	13	1	9	1	**24**
HIV-exposed	7	10	1	0	**18**
**Total**	**20**	**11**	**10**	**1**	**42**
**TC Newman**					
HIV-unexposed	13	0	16	0	**29**
HIV-exposed	0	0	1	0	**1**
**Total**	**13**	**0**	**17**	**0**	**30**
**Infants at 20-28 weeks (n=36)**					
**Mbekweni**					
HIV-unexposed	4	0	6	0	**10**
HIV-exposed	0	7	3	0	**10**
**Total**	**4**	**7**	**9**	**0**	**20**
**TC Newman**					
HIV-unexposed	2	0	14	0	**16**
HIV-exposed	0	0	0	0	**0**
**Total**	**2**	**0**	**14**	**0**	**16**

HIV: Human immunodeficiency virus

Supplementary Table S2. Faecal bacterial proportions from 90 mothers and infants with significantly distinct profiles at the time of delivery

	Infants at birth (n=90) Median (IQR)	Mothers at birth (n=90) Median (IQR)	p-value
**Phylum Proteobacteria**	**88.9 (72.8-96.2)**	3.2 (1.2-8.9)	<0.001
Alphaproteobacteria	**7.6 (0.5-24.3)**	0.1 (0.1-0.2)	<0.001
Caulobacterales	**2.9 (0.3-17.3)**	0.1 (0.1-0.1)	<0.001
Caulobacteraceae	**2.9 (0.3-17.3)**	0.1 (0.1-0.1)	<0.001
Brevundimonas	**2.0 (0.1-10.7)**	0.1 (0.0-0.1)	<0.001
OTU_15	**0.0 (0.0-1.9)**	0.0 (0.0-0.0)	<0.001
OTU_541	**0.4 (0.0-2.0)**	0.0 (0.0-0.0)	<0.001
Rhizobiales	**1.6 (0.1-3.9)**	0.0 (0.0-0.0)	<0.001
Rhizobiaceae	**0.2 (0.0-1.2)**	0.0 (0.0-0.0)	<0.001
Rhizobium	**0.2 (0.0-1.2)**	0.0 (0.0-0.0)	<0.001
Rhodobacterales	**0.2 (0.0-1.4)**	0.0 (0.0-0.0)	<0.001
Rhodobacteraceae	**0.2 (0.0-1.4)**	0.0 (0.0-0.0)	<0.001
Sphingomonadales	**0.8 (0.0-2.2)**	0.0 (0.0-0.0)	<0.001
Sphingomonadaceae	**0.7 (0.0-2.0)**	0.0 (0.0-0.0)	<0.001
Sphingomonas	**0.7 (0.0-1.9)**	0.0 (0.0-0.0)	<0.001
Betaproteobacteria	**15.9 (0.4-25.4)**	0.1 (0.1-0.2)	<0.001
Burkholderiales	**15.9 (0.4-25.0)**	0.1 (0.1-0.2)	<0.001
Comamonadaceae	**7.9 (0.2-17.0)**	0.1 (0.0-0.1)	<0.001
Comamonas	**1.5 (0.1-11.5)**	0.0 (0.0-0.1)	<0.001
OTU_14	**1.8 (0.0-3.8)**	0.0 (0.0-0.0)	<0.001
Oxalobacteraceae	**1.2 (0.1-7.7)**	0.0 (0.0-0.1)	<0.001
Massilia	**0.6 (0.0-3.4)**	0.0 (0.0-0.0)	<0.001
OTU_18	**0.2 (0.0-2.1)**	0.0 (0.0-0.0)	<0.001
Gammaproteobacteria	**45.4 (10.2-75.3)**	1.7 (0.8-7.4)	<0.001
aaa34a10	**0.1 (0.0-3.9)**	0.0 (0.0-0.0)	<0.001
OTU_11	**0.1 (0.0-3.9)**	0.0 (0.0-0.0)	<0.001
OTU_11	**0.1 (0.0-3.9)**	0.0 (0.0-0.0)	<0.001
Alteromonadales	**0.3 (0.1-8.8)**	0.0 (0.0-0.1)	<0.001
Shewanellaceae	**0.2 (0.1-8.6)**	0.0 (0.0-0.1)	<0.001
Shewanella	**0.2 (0.1-8.6)**	0.0 (0.0-0.1)	<0.001
Enterobacteriales	**1.3 (0.5-50.3)**	0.8 (0.4-3.6)	<0.001
Enterobacteriaceae	**1.3 (0.5-50.3)**	0.8 (0.4-3.6)	<0.001
Citrobacter	**0.0 (0.0-0.1)**	0.0 (0.0-0.0)	<0.001
Enterobacter	**0.2 (0.1-3.7)**	0.2 (0.1-1.0)	<0.001
Escherichia-Shigella	**0.7 (0.2-24.2)**	0.4 (0.3-2.2)	<0.001
OTU_101	**0.0 (0.0-0.1)**	0.0 (0.0-0.1)	<0.001
OTU_565	**0.0 (0.0-0.0)**	0.0 (0.0-0.0)	<0.001
OTU_615	**0.0 (0.0-0.1)**	0.0 (0.0-0.0)	<0.001
OTU_616	**0.0 (0.0-0.0)**	0.0 (0.0-0.0)	<0.001
Pseudomonadales	**5.1 (0.3-23.7)**	0.1 (0.1-0.2)	<0.001
Moraxellaceae	**3.6 (0.1-13.2)**	0.1 (0.0-0.1)	<0.001
Acinetobacter	**3.4 (0.1-12.2)**	0.1 (0.0-0.1)	<0.001
Pseudomonadaceae	**1.3 (0.1-10.8)**	0.0 (0.0-0.1)	<0.001
Pseudomonas	**1.3 (0.1-10.8)**	0.0 (0.0-0.1)	<0.001
Xanthomonadales	**0.3 (0.0-1.1)**	0.0 (0.0-0.0)	<0.001
Xanthomonadaceae	**0.3 (0.0-1.1)**	0.0 (0.0-0.0)	<0.001
**Phylum Firmicutes**	2.2 (0.9-16.8)	**66.4 (57.3-77.2)**	<0.001
Bacilli	**1.2 (0.5-4.6)**	1.0 (0.4-2.6)	0.0001
Bacillales	**0.2 (0.0-0.5)**	0.0 (0.0-0.1)	<0.001
Staphylococcaceae	**0.1 (0.0-0.3)**	0.0 (0.0-0.0)	<0.001
Staphylococcus	**0.1 (0.0-0.3)**	0.0 (0.0-0.0)	<0.001
Lactobacillales	0.7 (0.3-2.1)	**0.9 (0.4-2.6)**	<0.001
Streptococcaceae	**0.5 (0.2-1.3)**	0.3 (0.2-1.0)	<0.001
Streptococcus	**0.5 (0.2-1.3)**	0.3 (0.2-1.0)	<0.001
Clostridia	0.2 (0.1-1.7)	**49.2 (38.8-61.3)**	<0.001
Clostridiales	0.2 (0.1-1.7)	**49.2 (38.8-61.3)**	<0.001
Clostridiaceae 1	0.0 (0.0-0.1)	**2.8 (0.6-5.7)**	<0.001
Clostridium sensu stricto 1	0.0 (0.0-0.1)	**2.2 (0.6-4.9)**	<0.001
Family XIII	-	-	-
Incertae Sedis	0.1 (0.0-0.4)	**11.3 (6.8-14.3)**	<0.001

IQR: Interquartile range; OTU: Operational taxonomic unit

Bacterial proportions are rounded to one decimal point. Larger proportions of bacterial taxa are highlighted in bold.

Supplementary Table S2. Faecal bacterial proportions from 90 mothers and infants with significantly distinct profiles at the time of delivery (continued)

	**Infants at birth (n=90) Median (IQR)**	**Mothers at birth (n=90) Median (IQR)**	**p-value**
Lachnospiraceae	0.1 (0.0-0.2)	**20.1 (14.7-28.5)**	<0.001
Blautia	0.0 (0.0-0.0)	**5.2 (3.4-8.0)**	<0.001
OTU_19	0.0 (0.0-0.0)	**2.7 (1.8-4.0)**	<0.001
Peptostreptococcaceae	0.0 (0.0-0.1)	**2.7 (1.2-6.2)**	<0.001
Ruminococcaceae	0.0 (0.0-0.1)	**17.0 (12.5-23.1)**	<0.001
Faecalibacterium	0.0 (0.0-0.0)	**7.0 (3.6-10.8)**	<0.001
Ruminococcus	0.0 (0.0-0.0)	**2.5 (1.3-5.6)**	<0.001
Subdoligranulum	0.0 (0.0-0.0)	**1.8 (1.0-3.2)**	<0.001
Erysipelotrichia	0.0 (0.0-0.1)	**11.1 (7.1-13.9)**	<0.001
Erysipelotrichales	0.0 (0.0-0.2)	**11.1 (7.1-13.9)**	<0.001
Erysipelotrichaceae	0.0 (0.0-0.2)	**11.1 (7.1-13.9)**	<0.001
Catenibacterium	0.0 (0.0-0.0)	**4.6 (1.6-9.9)**	<0.001
**Phylum Actinobacteria**	4.0 (1.7-10.4)	**14.5 (6.4-24.5)**	<0.001
Actinobacteria	3.7 (1.6-10.2)	**11.2 (3.3-21.6)**	<0.001
Bifidobacteriales	0.3 (0.2-0.8)	**10.8 (3.3-21.6)**	<0.001
Bifidobacteriaceae	0.3 (0.2-0.8)	**10.8 (3.3-21.6)**	<0.001
Bifidobacterium	0.3 (0.2-0.8)	**10.8 (3.3-21.6)**	<0.001
Coriobacteriia	0.0 (0.0-0.1)	**2.1 (1.4-3.3)**	<0.001
Coriobacteriales	0.0 (0.0-0.1)	**2.1 (1.4-3.3)**	<0.001
Coriobacteriaceae	0.0 (0.0-0.1)	**2.1 (1.4-3.3)**	<0.001
Collinsella	0.0 (0.0-0.0)	**1.5 (0.8-2.2)**	<0.001
Frankiales	**0.5 (0.0-1.7)**	0.0 (0.0-0.0)	<0.001
Geodermatophilaceae	**0.5 (0.0-1.6)**	0.0 (0.0-0.0)	<0.001
Blastococcus	**0.5 (0.0-1.6)**	0.0 (0.0-0.0)	<0.001
Micrococcales	**0.8 (0.0-1.8)**	0.0 (0.0-0.0)	<0.001
Micrococcaceae	**0.7 (0.0-1.8)**	0.0 (0.0-0.0)	<0.001
Renibacterium	**0.7 (0.0-1.8)**	0.0 (0.0-0.0)	<0.001
**Phylum Bacteroidetes**	0.2 (0.0-0.7)	**5.0 (1.7-12.3)**	<0.001
Bacteroidia	0.0 (0.0-0.1)	**5.0 (1.7-12.3)**	<0.001
Bacteroidales	0.0 (0.0-0.1)	**5.0 (1.7-12.3)**	<0.001
Prevotellaceae	0.0 (0.0-0.1)	**1.6 (0.3-7.9)**	<0.001
Prevotella	0.0 (0.0-0.0)	**1.5 (0.2-7.0)**	<0.001

IQR: Interquartile range; OTU: Operational taxonomic unit
Bacterial proportions are rounded to one decimal point. Larger proportions of bacterial taxa are highlighted in bold.

Supplementary Table S3. Faecal bacterial proportions observed from maternal and infant faecal specimens (n=305) included in the study

	% of bacteria			
	**Infants at birth (n=107)**	**Infants at 4 to 12 weeks (n=72)**	**Infants at 20-28 weeks (n=36)**	**Mothers at birth (n=90)**
**Phylum-level**				
Actinobacteria	**6.8059**	**60.2713**	**52.8940**	**16.3161**
Firmicutes	**13.3498**	**21.3879**	**32.9042**	**65.2749**
Proteobacteria	**78.0889**	**15.8279**	**11.2723**	**8.2932**
Bacteroidetes	**1.1384**	**2.3141**	**2.5159**	**8.5418**
Verrucomicrobia	0.0033	0.0022	0.1163	**0.7881**
Euryarchaeota	0.0137	0.0006	0.0033	0.3094
Deinococcus-Thermus	0.1153	0.0006	0.0018	0.0014
OTU_174	0.0266	0.0181	0.0220	0.0308
Cyanobacteria	0.0567	0.0001	0.0000	0.0344
OTU_363	0.0042	0.0022	0.0550	0.0133
OTU_151	0.0209	0.0135	0.0187	0.0188
OTU_236	0.0161	0.0127	0.0147	0.0148
Tenericutes	0.0144	0.0000	0.0065	0.0354
OTU_184	0.0149	0.0103	0.0104	0.0147
Candidate division TM7	0.0480	0.0002	0.0000	0.0002
OTU_182	0.0122	0.0099	0.0124	0.0138
OTU_185	0.0140	0.0111	0.0056	0.0160
OTU_161	0.0131	0.0096	0.0084	0.0153
OTU_303	0.0108	0.0085	0.0122	0.0145
OTU_290	0.0141	0.0079	0.0092	0.0133
OTU_204	0.0092	0.0102	0.0077	0.0165
OTU_287	0.0117	0.0055	0.0062	0.0158
OTU_190	0.0081	0.0084	0.0093	0.0112
OTU_314	0.0111	0.0079	0.0078	0.0099
Fusobacteria	0.0106	0.0000	0.0000	0.0255
OTU_417	0.0342	0.0000	0.0000	0.0006
OTU_252	0.0344	0.0000	0.0000	0.0001
OTU_327	0.0118	0.0041	0.0070	0.0097
OTU_465	0.0003	0.0000	0.0000	0.0304
OTU_545	0.0001	0.0001	0.0000	0.0300
OTU_265	0.0062	0.0052	0.0087	0.0087
OTU_278	0.0069	0.0050	0.0066	0.0099
OTU_341	0.0039	0.0047	0.0088	0.0068
OTU_234	0.0076	0.0043	0.0078	0.0040
OTU_259	0.0055	0.0034	0.0062	0.0073
OTU_280	0.0058	0.0050	0.0073	0.0041
OTU_350	0.0052	0.0038	0.0049	0.0076
OTU_263	0.0043	0.0032	0.0045	0.0082
OTU_332	0.0059	0.0043	0.0034	0.0047
OTU_313	0.0059	0.0021	0.0044	0.0057
OTU_351	0.0069	0.0026	0.0022	0.0060
OTU_364	0.0042	0.0013	0.0036	0.0065
OTU_289	0.0045	0.0026	0.0029	0.0044
OTU_452	0.0045	0.0026	0.0028	0.0025
OTU_398	0.0026	0.0023	0.0041	0.0021
OTU_358	0.0038	0.0029	0.0008	0.0012
Chloroflexi	0.0022	0.0000	0.0000	0.0000
OTU_602	0.0014	0.0000	0.0000	0.0000
**Class-level**				
Actinobacteria	**6.4437**	**58.1842**	**50.1228**	**13.1906**
Gammaproteobacteria	**46.7729**	**15.6906**	**11.0728**	**7.3580**
Clostridia	**4.5215**	**4.0252**	**13.8298**	**50.1438**
Bacilli	**7.4922**	**14.8418**	**16.6740**	**2.6560**
Alphaproteobacteria	**16.4449**	0.0784	0.0847	0.1639
Erysipelotrichia	**1.1790**	**1.8852**	**1.6759**	**11.3978**
Betaproteobacteria	**14.7879**	0.0541	0.0822	0.1837
Bacteroidia	**0.9298**	**2.3133**	**2.5139**	**8.5382**
Coriobacteriia	0.3600	**2.0871**	**2.7711**	**3.1256**
Negativicutes	0.1300	**0.6358**	**0.7245**	**1.0764**
Verrucomicrobiae	0.0033	0.0022	0.1163	**0.7881**
Deltaproteobacteria	0.0754	0.0045	0.0019	**0.5877**
Methanobacteria	0.0137	0.0006	0.0033	0.3094
Deinococci	0.1153	0.0006	0.0018	0.0014
Flavobacteriia	0.1087	0.0005	0.0016	0.0028

OTU: Operational taxonomic unit
Bacterial proportions >0.5% are highlighted in bold

Supplementary Table S3. Faecal bacterial proportions observed from maternal and infant faecal specimens (n=305) included in the study (continued)

	% of bacteria			
	**Infants at birth (n=107)**	**Infants at 4 to 12 weeks (n=72)**	**Infants at 20-28 weeks (n=36)**	**Mothers at birth (n=90)**
OTU_174	0.0266	0.0181	0.0220	0.0308
OTU_363	0.0042	0.0022	0.0550	0.0133
OTU_151	0.0209	0.0135	0.0187	0.0188
OTU_236	0.0161	0.0127	0.0147	0.0148
Mollicutes	0.0144	0.0000	0.0065	0.0354
Cytophagia	0.0518	0.0000	0.0000	0.0000
Chloroplast	0.0499	0.0001	0.0000	0.0015
OTU_184	0.0149	0.0103	0.0104	0.0147
Sphingobacteriia	0.0482	0.0003	0.0004	0.0008
OTU_182	0.0122	0.0099	0.0124	0.0138
OTU_185	0.0140	0.0111	0.0056	0.0160
OTU_161	0.0131	0.0096	0.0084	0.0153
OTU_303	0.0108	0.0085	0.0122	0.0145
OTU_290	0.0141	0.0079	0.0092	0.0133
OTU_204	0.0092	0.0102	0.0077	0.0165
OTU_287	0.0117	0.0055	0.0062	0.0158
Epsilonproteobacteria	0.0077	0.0003	0.0307	0.0000
OTU_190	0.0081	0.0084	0.0093	0.0112
OTU_314	0.0111	0.0079	0.0078	0.0099
Fusobacteriia	0.0106	0.0000	0.0000	0.0255
OTU_417	0.0342	0.0000	0.0000	0.0006
OTU_252	0.0344	0.0000	0.0000	0.0001
Melainabacteria	0.0000	0.0000	0.0000	0.0329
OTU_327	0.0118	0.0041	0.0070	0.0097
OTU_465	0.0003	0.0000	0.0000	0.0304
OTU_545	0.0001	0.0001	0.0000	0.0300
OTU_265	0.0062	0.0052	0.0087	0.0087
OTU_278	0.0069	0.0050	0.0066	0.0099
OTU_337	0.0271	0.0000	0.0000	0.0009
OTU_300	0.0250	0.0000	0.0000	0.0000
OTU_341	0.0039	0.0047	0.0088	0.0068
OTU_234	0.0076	0.0043	0.0078	0.0040
OTU_259	0.0055	0.0034	0.0062	0.0073
OTU_280	0.0058	0.0050	0.0073	0.0041
OTU_350	0.0052	0.0038	0.0049	0.0076
OTU_263	0.0043	0.0032	0.0045	0.0082
OTU_574	0.0184	0.0000	0.0000	0.0002
OTU_332	0.0059	0.0043	0.0034	0.0047
OTU_313	0.0059	0.0021	0.0044	0.0057
OTU_351	0.0069	0.0026	0.0022	0.0060
OTU_364	0.0042	0.0013	0.0036	0.0065
OTU_289	0.0045	0.0026	0.0029	0.0044
OTU_452	0.0045	0.0026	0.0028	0.0025
OTU_398	0.0026	0.0023	0.0041	0.0021
OTU_358	0.0038	0.0029	0.0008	0.0012
ML635J-21	0.0068	0.0000	0.0000	0.0000
OTU_373	0.0027	0.0002	0.0000	0.0000
Thermoleophilia	0.0022	0.0000	0.0000	0.0000
Thermomicrobia	0.0017	0.0000	0.0000	0.0000
OTU_602	0.0014	0.0000	0.0000	0.0000
OTU_587	0.0014	0.0000	0.0000	0.0000
uncultured soil bacterium	0.0006	0.0000	0.0000	0.0000
Caldilineae	0.0005	0.0000	0.0000	0.0000
**Order-level**				
Bifidobacteriales	**2.5302**	**58.1207**	**50.0215**	**13.1144**
Clostridiales	**4.5215**	**4.0252**	**13.8298**	**50.1438**
Enterobacteriales	**24.4464**	**15.5511**	**10.9039**	**4.8390**
Lactobacillales	**4.7619**	**14.7745**	**16.6279**	**2.5993**
Erysipelotrichales	**1.1790**	**1.8852**	**1.6759**	**11.3978**
Pseudomonadales	**14.2852**	0.0525	0.0629	**0.5553**
Burkholderiales	**14.6158**	0.0538	0.0822	0.1810
Bacteroidales	**0.9298**	**2.3133**	**2.5139**	**8.5382**
Caulobacterales	**10.7038**	0.0531	0.0565	0.1057
Coriobacteriales	0.3600	**2.0871**	**2.7711**	**3.1256**

OTU: Operational taxonomic unit
Bacterial proportions >0.5% are highlighted in bold

Supplementary Table S3. Faecal bacterial proportions observed from maternal and infant faecal specimens (n=305) included in the study (continued)

	% of bacteria			
	**Infants at birth (n=107)**	**Infants at 4 to 12 weeks (n=72)**	**Infants at 20-28 weeks (n=36)**	**Mothers at birth (n=90)**
Alteromonadales	**4.7978**	0.0272	0.0233	0.0663
Bacillales	**2.7303**	0.0673	0.0462	0.0567
Rhizobiales	**2.5254**	0.0126	0.0153	0.0259
Selenomonadales	0.1300	**0.6358**	**0.7245**	**1.0764**
Frankiales	**2.1252**	0.0062	0.0083	0.0306
Aeromonadales	0.1395	0.0025	0.0027	**1.8184**
aaa34a10	**1.7414**	0.0079	0.0068	0.0197
Sphingomonadales	**1.4083**	0.0055	0.0027	0.0144
Micrococcales	**1.2563**	0.0465	0.0493	0.0233
Xanthomonadales	**1.1348**	0.0045	0.0027	0.0077
Verrucomicrobiales	0.0033	0.0022	0.1163	**0.7881**
Rhodobacterales	**0.8743**	0.0044	0.0052	0.0073
Desulfovibrionales	0.0251	0.0045	0.0019	**0.5868**
OTU_112	**0.5249**	0.0008	0.0026	0.0039
Rhodospirillales	0.3763	0.0015	0.0019	0.0066
Corynebacteriales	0.2935	0.0043	0.0284	0.0029
Methanobacteriales	0.0137	0.0006	0.0033	0.3094
Propionibacteriales	0.2328	0.0008	0.0010	0.0022
Pasteurellales	0.0993	0.0014	0.0414	0.0414
Neisseriales	0.1515	0.0003	0.0000	0.0022
Deinococcales	0.1153	0.0006	0.0018	0.0014
Flavobacteriales	0.1087	0.0005	0.0016	0.0028
OTU_594	0.0176	0.0428	0.0291	0.0091
OTU_174	0.0266	0.0181	0.0220	0.0308
Chromatiales	0.0902	0.0006	0.0000	0.0011
OTU_363	0.0042	0.0022	0.0550	0.0133
OTU_151	0.0209	0.0135	0.0187	0.0188
OTU_236	0.0161	0.0127	0.0147	0.0148
Cytophagales	0.0518	0.0000	0.0000	0.0000
OTU_171	0.0499	0.0001	0.0000	0.0015
Bdellovibrionales	0.0503	0.0000	0.0000	0.0010
OTU_184	0.0149	0.0103	0.0104	0.0147
Sphingobacteriales	0.0482	0.0003	0.0004	0.0008
OTU_182	0.0122	0.0099	0.0124	0.0138
RF9	0.0058	0.0000	0.0065	0.0354
OTU_185	0.0140	0.0111	0.0056	0.0160
OTU_161	0.0131	0.0096	0.0084	0.0153
OTU_303	0.0108	0.0085	0.0122	0.0145
OTU_290	0.0141	0.0079	0.0092	0.0133
OTU_204	0.0092	0.0102	0.0077	0.0165
Actinomycetales	0.0058	0.0057	0.0143	0.0171
OTU_287	0.0117	0.0055	0.0062	0.0158
Campylobacterales	0.0077	0.0003	0.0307	0.0000
OTU_190	0.0081	0.0084	0.0093	0.0112
OTU_314	0.0111	0.0079	0.0078	0.0099
Fusobacteriales	0.0106	0.0000	0.0000	0.0255
OTU_417	0.0342	0.0000	0.0000	0.0006
OTU_252	0.0344	0.0000	0.0000	0.0001
Gastranaerophilales	0.0000	0.0000	0.0000	0.0329
OTU_327	0.0118	0.0041	0.0070	0.0097
OTU_465	0.0003	0.0000	0.0000	0.0304
OTU_545	0.0001	0.0001	0.0000	0.0300
DB1-14	0.0284	0.0006	0.0000	0.0000
OTU_265	0.0062	0.0052	0.0087	0.0087
OTU_278	0.0069	0.0050	0.0066	0.0099
OTU_337	0.0271	0.0000	0.0000	0.0009
OTU_300	0.0250	0.0000	0.0000	0.0000
OTU_341	0.0039	0.0047	0.0088	0.0068
OTU_234	0.0076	0.0043	0.0078	0.0040
OTU_259	0.0055	0.0034	0.0062	0.0073
OTU_280	0.0058	0.0050	0.0073	0.0041
OTU_350	0.0052	0.0038	0.0049	0.0076
Hydrogenophilales	0.0206	0.0000	0.0000	0.0004
OTU_263	0.0043	0.0032	0.0045	0.0082
OTU_574	0.0184	0.0000	0.0000	0.0002

OTU: Operational taxonomic unit
Bacterial proportions >0.5% are highlighted in bold

Supplementary Table S3. Faecal bacterial proportions observed from maternal and infant faecal specimens (n=305) included in the study (continued)

	% of bacteria			
	**Infants at birth (n=107)**	**Infants at 4 to 12 weeks (n=72)**	**Infants at 20-28 weeks (n=36)**	**Mothers at birth (n=90)**
OTU_332	0.0059	0.0043	0.0034	0.0047
OTU_313	0.0059	0.0021	0.0044	0.0057
OTU_351	0.0069	0.0026	0.0022	0.0060
OTU_364	0.0042	0.0013	0.0036	0.0065
OTU_289	0.0045	0.0026	0.0029	0.0044
Vibrionales	0.0135	0.0000	0.0000	0.0000
OTU_452	0.0045	0.0026	0.0028	0.0025
OTU_398	0.0026	0.0023	0.0041	0.0021
OTU_358	0.0038	0.0029	0.0008	0.0012
Mycoplasmatales	0.0086	0.0000	0.0000	0.0000
OTU_473	0.0068	0.0000	0.0000	0.0000
Legionellales	0.0040	0.0000	0.0000	0.0000
Rickettsiales	0.0034	0.0000	0.0005	0.0000
NKB5	0.0031	0.0000	0.0000	0.0000
OTU_373	0.0027	0.0002	0.0000	0.0000
Gaiellales	0.0022	0.0000	0.0000	0.0000
JG30-KF-CM45	0.0017	0.0000	0.0000	0.0000
OTU_602	0.0014	0.0000	0.0000	0.0000
OTU_587	0.0014	0.0000	0.0000	0.0000
uncultured soil bacterium	0.0006	0.0000	0.0000	0.0000
Caldilineales	0.0005	0.0000	0.0000	0.0000
Myxococcales	0.0000	0.0000	0.0000	0.0000
**Family-level**				
Bifidobacteriaceae	**2.5302**	**58.1207**	**50.0215**	**13.1144**
Enterobacteriaceae	**24.4464**	**15.5511**	**10.9039**	**4.8390**
Lachnospiraceae	**0.9361**	**3.5397**	**11.7223**	**22.3670**
Streptococcaceae	**3.5846**	**9.3642**	**9.9516**	**1.2987**
Ruminococcaceae	**0.7950**	0.1239	**1.0157**	**17.8557**
Erysipelotrichaceae	**1.1790**	**1.8852**	**1.6759**	**11.3978**
Caulobacteraceae	**10.7038**	0.0531	0.0565	0.1057
Comamonadaceae	**9.7601**	0.0304	0.0286	0.0944
Moraxellaceae	**8.4793**	0.0331	0.0413	0.4862
Prevotellaceae	0.4664	**1.2768**	0.3133	**6.4422**
Coriobacteriaceae	0.3600	**2.0871**	**2.7711**	**3.1256**
Lactobacillaceae	0.4241	**2.0976**	**2.9537**	**1.1572**
Peptostreptococcaceae	**1.2464**	0.0745	**0.5215**	**4.2925**
Leuconostocaceae	0.1791	**2.6196**	**3.1821**	0.0386
Pseudomonadaceae	**5.8059**	0.0194	0.0217	0.0692
Clostridiaceae 1	**0.9148**	0.0904	0.3545	**4.4276**
Shewanellaceae	**4.7779**	0.0269	0.0233	0.0663
Oxalobacteraceae	**4.7038**	0.0160	0.0143	0.0349
Bacteroidaceae	0.3178	**0.9064**	**2.1360**	**1.3752**
Veillonellaceae	0.1140	**0.6281**	**0.7214**	**0.9905**
Geodermatophilaceae	**2.1216**	0.0062	0.0083	0.0306
Succinivibrionaceae	0.1395	0.0025	0.0027	**1.8184**
Staphylococcaceae	**1.8058**	0.0635	0.0405	0.0445
Enterococcaceae	**0.5668**	**0.6931**	**0.5405**	0.1048
OTU_11	**1.7414**	0.0079	0.0068	0.0197
Micrococcaceae	**1.2479**	0.0465	0.0493	0.0233
Sphingomonadaceae	**1.3193**	0.0053	0.0027	0.0134
Xanthomonadaceae	**1.1335**	0.0045	0.0027	0.0077
Verrucomicrobiaceae	0.0033	0.0022	0.1163	**0.7881**
Bacillaceae	**0.8813**	0.0037	0.0056	0.0120
Rhodobacteraceae	**0.8743**	0.0044	0.0052	0.0073
Rhizobiaceae	**0.7896**	0.0049	0.0057	0.0091
OTU_345	0.1000	0.1672	0.1776	0.2856
Desulfovibrionaceae	0.0251	0.0045	0.0019	**0.5868**
Porphyromonadaceae	0.1195	0.1283	0.0628	0.2422
OTU_112	**0.5249**	0.0008	0.0026	0.0039
Aurantimonadaceae	**0.5184**	0.0041	0.0037	0.0027
Methylocystaceae	0.4989	0.0018	0.0028	0.0030
OTU_137	0.3466	0.0041	0.0131	0.1112
Methanobacteriaceae	0.0137	0.0006	0.0033	0.3094
Corynebacteriaceae	0.2766	0.0043	0.0284	0.0019

OTU: Operational taxonomic unit
Bacterial proportions >0.5% are highlighted in bold

Supplementary Table S3. Faecal bacterial proportions observed from maternal and infant faecal specimens (n=305) included in the study (continued)

	% of bacteria			
	**Infants at birth (n=107)**	**Infants at 4 to 12 weeks (n=72)**	**Infants at 20-28 weeks (n=36)**	**Mothers at birth (n=90)**
Rhodospirillaceae	0.3007	0.0011	0.0015	0.0058
Peptococcaceae	0.0200	0.0002	0.0000	0.2823
Rikenellaceae	0.0049	0.0013	0.0018	0.2874
Hyphomicrobiaceae	0.2854	0.0011	0.0024	0.0051
Christensenellaceae	0.0179	0.0000	0.0028	0.2626
Nocardioidaceae	0.2328	0.0008	0.0010	0.0022
Family XIII	0.0186	0.0031	0.0019	0.1992
S24-7	0.0212	0.0006	0.0000	0.1912
Bradyrhizobiaceae	0.1957	0.0002	0.0000	0.0025
Pasteurellaceae	0.0993	0.0014	0.0414	0.0414
Methylobacteriaceae	0.1766	0.0005	0.0002	0.0027
Neisseriaceae	0.1515	0.0003	0.0000	0.0022
Family XI	0.1240	0.0115	0.0045	0.0104
Burkholderiaceae	0.1258	0.0005	0.0011	0.0015
Alcaligenaceae	0.0261	0.0070	0.0382	0.0502
Deinococcaceae	0.1153	0.0006	0.0018	0.0014
Flavobacteriaceae	0.1087	0.0005	0.0016	0.0028
Acidaminococcaceae	0.0159	0.0076	0.0031	0.0859
OTU_594	0.0176	0.0428	0.0291	0.0091
OTU_174	0.0266	0.0181	0.0220	0.0308
Chromatiaceae	0.0902	0.0006	0.0000	0.0011
Erythrobacteraceae	0.0810	0.0002	0.0000	0.0010
Acetobacteraceae	0.0756	0.0004	0.0004	0.0008
OTU_363	0.0042	0.0022	0.0550	0.0133
OTU_151	0.0209	0.0135	0.0187	0.0188
OTU_236	0.0161	0.0127	0.0147	0.0148
OTU_171	0.0499	0.0001	0.0000	0.0015
OTU_184	0.0149	0.0103	0.0104	0.0147
Chitinophagaceae	0.0482	0.0003	0.0004	0.0008
OTU_182	0.0122	0.0099	0.0124	0.0138
Cytophagaceae	0.0474	0.0000	0.0000	0.0000
OTU_185	0.0140	0.0111	0.0056	0.0160
OTU_161	0.0131	0.0096	0.0084	0.0153
OTU_303	0.0108	0.0085	0.0122	0.0145
OTU_290	0.0141	0.0079	0.0092	0.0133
Xanthobacteraceae	0.0433	0.0000	0.0006	0.0003
OTU_204	0.0092	0.0102	0.0077	0.0165
Actinomycetaceae	0.0058	0.0057	0.0143	0.0171
OTU_441	0.0012	0.0000	0.0000	0.0401
OTU_287	0.0117	0.0055	0.0062	0.0158
OTU_190	0.0081	0.0084	0.0093	0.0112
OTU_314	0.0111	0.0079	0.0078	0.0099
Fusobacteriaceae	0.0106	0.0000	0.0000	0.0255
OTU_417	0.0342	0.0000	0.0000	0.0006
OTU_252	0.0344	0.0000	0.0000	0.0001
OTU_440	0.0000	0.0000	0.0000	0.0329
OTU_327	0.0118	0.0041	0.0070	0.0097
Helicobacteraceae	0.0001	0.0000	0.0307	0.0000
OTU_465	0.0003	0.0000	0.0000	0.0304
OTU_545	0.0001	0.0001	0.0000	0.0300
Bdellovibrionaceae	0.0291	0.0000	0.0000	0.0006
Eubacteriaceae	0.0004	0.0107	0.0160	0.0016
OTU_265	0.0062	0.0052	0.0087	0.0087
OTU_278	0.0069	0.0050	0.0066	0.0099
OTU_337	0.0271	0.0000	0.0000	0.0009
OTU_617	0.0005	0.0000	0.0065	0.0185
OTU_300	0.0250	0.0000	0.0000	0.0000
OTU_341	0.0039	0.0047	0.0088	0.0068
OTU_234	0.0076	0.0043	0.0078	0.0040
Planococcaceae	0.0230	0.0000	0.0000	0.0002
OTU_259	0.0055	0.0034	0.0062	0.0073
OTU_280	0.0058	0.0050	0.0073	0.0041
OTU_564	0.0053	0.0000	0.0000	0.0169
Bacteriovoracaceae	0.0212	0.0000	0.0000	0.0004
OTU_350	0.0052	0.0038	0.0049	0.0076
Hydrogenophilaceae	0.0206	0.0000	0.0000	0.0004

OTU: Operational taxonomic unit
Bacterial proportions >0.5% are highlighted in bold

Supplementary Table S3. Faecal bacterial proportions observed from maternal and infant faecal specimens (n=305) included in the study (continued)

	% of bacteria			
	**Infants at birth (n=107)**	**Infants at 4 to 12 weeks (n=72)**	**Infants at 20-28 weeks (n=36)**	**Mothers at birth (n=90)**
OTU_263	0.0043	0.0032	0.0045	0.0082
OTU_257	0.0201	0.0001	0.0000	0.0000
OTU_574	0.0184	0.0000	0.0000	0.0002
OTU_332	0.0059	0.0043	0.0034	0.0047
OTU_223	0.0176	0.0000	0.0000	0.0005
OTU_313	0.0059	0.0021	0.0044	0.0057
OTU_351	0.0069	0.0026	0.0022	0.0060
OTU_364	0.0042	0.0013	0.0036	0.0065
Idiomarinaceae	0.0152	0.0003	0.0000	0.0000
Dietziaceae	0.0136	0.0000	0.0000	0.0009
OTU_289	0.0045	0.0026	0.0029	0.0044
Vibrionaceae	0.0135	0.0000	0.0000	0.0000
OTU_522	0.0124	0.0006	0.0000	0.0000
OTU_452	0.0045	0.0026	0.0028	0.0025
OTU_398	0.0026	0.0023	0.0041	0.0021
OTU_358	0.0038	0.0029	0.0008	0.0012
Mycoplasmataceae	0.0086	0.0000	0.0000	0.0000
Dermabacteraceae	0.0084	0.0000	0.0000	0.0000
Defluviitaleaceae	0.0005	0.0000	0.0000	0.0079
uncultured gamma proteobacterium	0.0082	0.0000	0.0000	0.0000
7B-8	0.0080	0.0000	0.0000	0.0000
Campylobacteraceae	0.0076	0.0003	0.0000	0.0000
uncultured alpha proteobacterium	0.0078	0.0000	0.0000	0.0000
Aerococcaceae	0.0072	0.0000	0.0000	0.0000
OTU_473	0.0068	0.0000	0.0000	0.0000
Alteromonadaceae	0.0048	0.0000	0.0000	0.0000
Cyclobacteriaceae	0.0044	0.0000	0.0000	0.0000
Coxiellaceae	0.0040	0.0000	0.0000	0.0000
mitochondria	0.0034	0.0000	0.0005	0.0000
uncultured actinobacterium	0.0036	0.0000	0.0000	0.0000
Mycobacteriaceae	0.0033	0.0000	0.0000	0.0000
OTU_488	0.0031	0.0000	0.0000	0.0000
OTU_373	0.0027	0.0002	0.0000	0.0000
OTU_576	0.0022	0.0000	0.0000	0.0000
OTU_542	0.0017	0.0000	0.0000	0.0000
OTU_602	0.0014	0.0000	0.0000	0.0000
OTU_587	0.0014	0.0000	0.0000	0.0000
OTU_552	0.0013	0.0000	0.0000	0.0000
uncultured soil bacterium	0.0006	0.0000	0.0000	0.0000
Caldilineaceae	0.0005	0.0000	0.0000	0.0000
Nannocystaceae	0.0000	0.0000	0.0000	0.0000
**Genus-level**				
Bifidobacterium	**2.5302**	**58.1207**	**50.0215**	**13.1144**
Escherichia-Shigella	**12.4631**	**8.3827**	**5.8453**	**2.7270**
Incertae Sedis	**1.3252**	**3.5205**	**7.1959**	**11.4539**
Streptococcus	**3.5805**	**9.0589**	**8.5477**	**1.2778**
Enterobacter	**5.8249**	**4.1146**	**2.6466**	**1.3472**
Blautia	0.2812	0.3587	**2.1772**	**5.9529**
Acinetobacter	**8.1086**	0.0310	0.0397	0.4812
Prevotella	0.4291	**1.2748**	0.3122	**6.0682**
Faecalibacterium	0.2076	0.0577	0.4546	**7.3397**
Catenibacterium	**0.6804**	0.4027	0.2969	**6.3585**
Brevundimonas	**6.6693**	0.0323	0.0347	0.0648
Lactobacillus	0.4241	**2.0976**	**2.9537**	**1.1572**
Collinsella	0.2945	**1.6295**	**2.6637**	**2.0315**
Comamonas	**6.2055**	0.0133	0.0141	0.0648
Pseudomonas	**5.8026**	0.0194	0.0217	0.0692
Weissella	0.1765	**2.5280**	**2.6803**	0.0358
Clostridium sensu stricto 1	**0.8845**	0.0883	0.3375	**3.6548**
Shewanella	**4.7779**	0.0269	0.0233	0.0663
Bacteroides	0.3178	**0.9064**	**2.1360**	**1.3752**
OTU_19	0.1604	0.2404	**1.0401**	**3.1166**
Ruminococcus	0.2468	0.0046	0.1446	**4.0383**
OTU_101	**1.8670**	**1.0363**	**0.6657**	0.2159

OTU: Operational taxonomic unit

Bacterial proportions >0.5% are highlighted in bold

Supplementary Table S3. Faecal bacterial proportions observed from maternal and infant faecal specimens (n=305) included in the study (continued)

	% of bacteria			
	**Infants at birth (n=107)**	**Infants at 4 to 12 weeks (n=72)**	**Infants at 20-28 weeks (n=36)**	**Mothers at birth (n=90)**
Subdoligranulum	0.0977	0.0296	0.2569	**2.6319**
Citrobacter	**1.2323**	**0.6551**	**0.5992**	0.1731
OTU_615	**1.0865**	**0.5698**	0.4728	0.1319
Blastococcus	**2.0977**	0.0062	0.0083	0.0301
OTU_50	0.0461	0.0213	0.3163	**1.7547**
Massilia	**2.0318**	0.0047	0.0035	0.0157
OTU_14	**1.9887**	0.0122	0.0085	0.0170
Staphylococcus	**1.8058**	0.0635	0.0405	0.0445
Enterococcus	**0.5668**	**0.6931**	**0.5405**	0.1048
OTU_11	**1.7414**	0.0079	0.0068	0.0197
OTU_35	0.0853	0.1325	0.1162	**1.4140**
Lactococcus	0.0041	0.3053	**1.4039**	0.0209
Roseburia	0.0377	0.0154	0.1484	**1.4936**
OTU_40	0.2481	0.0040	0.0625	**1.3559**
OTU_338	0.0044	0.2126	**1.3972**	0.0369
OTU_616	**0.7980**	0.4049	0.2961	0.1029
OTU_541	**1.5474**	0.0061	0.0056	0.0118
Sphingomonas	**1.2877**	0.0053	0.0027	0.0130
Renibacterium	**1.1928**	0.0063	0.0038	0.0110
OTU_18	**1.1881**	0.0047	0.0063	0.0083
OTU_15	**1.1385**	0.0077	0.0075	0.0134
OTU_55	0.0774	0.0446	0.1937	**0.8230**
Turicibacter	0.0381	0.0025	0.0127	**1.0811**
OTU_565	**0.5101**	0.2391	0.2641	0.0821
Megasphaera	0.0758	0.2620	0.4457	0.2278
Akkermansia	0.0033	0.0022	0.1163	**0.7881**
Bacillus	**0.8813**	0.0037	0.0056	0.0120
Succinivibrio	0.0554	0.0011	0.0015	**0.8246**
Sarcina	0.0302	0.0021	0.0169	**0.7728**
Rhizobium	**0.7896**	0.0049	0.0057	0.0091
Caulobacter	**0.7453**	0.0039	0.0037	0.0078
OTU_345	0.1000	0.1672	0.1776	0.2856
OTU_71	0.0504	0.0012	0.0000	**0.6432**
Coprococcus	0.0220	0.0201	0.0749	**0.5635**
OTU_212	**0.6617**	0.0019	0.0010	0.0026
Dialister	0.0197	0.0021	0.0108	**0.6299**
Serratia	**0.5328**	0.0510	0.0455	0.0285
Aquabacterium	**0.5948**	0.0025	0.0009	0.0037
OTU_555	**0.5908**	0.0035	0.0015	0.0058
Lysobacter	**0.5911**	0.0031	0.0007	0.0056
OTU_88	0.0437	0.0024	0.0902	0.4574
Acidovorax	**0.5687**	0.0004	0.0017	0.0062
Desulfovibrio	0.0248	0.0027	0.0009	**0.5336**
Parabacteroides	0.1195	0.1283	0.0628	0.2422
OTU_112	**0.5249**	0.0008	0.0026	0.0039
Veillonella	0.0137	0.3615	0.1179	0.0193
Anaerostipes	0.0087	0.0117	0.1942	0.2964
Pleomorphomonas	0.4989	0.0018	0.0028	0.0030
OTU_98	0.0412	0.0032	0.0024	0.4480
OTU_137	0.3466	0.0041	0.0131	0.1112
OTU_65	0.0449	0.0012	0.0009	0.4229
OTU_45	0.4558	0.0023	0.0039	0.0031
Fructobacillus	0.0019	0.0319	0.3959	0.0028
OTU_75	0.4185	0.0039	0.0027	0.0020
Dorea	0.0264	0.0005	0.0510	0.3338
OTU_301	0.0016	0.0754	0.0756	0.2569
OTU_442	0.0336	0.0003	0.0012	0.3506
OTU_232	0.0013	0.1812	0.1848	0.0156
Alloprevotella	0.0330	0.0020	0.0011	0.3105
OTU_188	0.0086	0.0002	0.0026	0.3048
OTU_135	0.0177	0.0002	0.0007	0.2948
Corynebacterium	0.2766	0.0043	0.0284	0.0019
OTU_99	0.0328	0.0010	0.0011	0.2745
Peptococcus	0.0200	0.0002	0.0000	0.2823
OTU_79	0.0009	0.1697	0.1272	0.0023

OTU: Operational taxonomic unit

Bacterial proportions >0.5% are highlighted in bold

Supplementary Table S3. Faecal bacterial proportions observed from maternal and infant faecal specimens (n=305) included in the study (continued)

	% of bacteria			
	**Infants at birth (n=107)**	**Infants at 4 to 12 weeks (n=72)**	**Infants at 20-28 weeks (n=36)**	**Mothers at birth (n=90)**
Devosia	0.2854	0.0011	0.0024	0.0051
OTU_80	0.0180	0.1410	0.0045	0.1301
OTU_52	0.2895	0.0005	0.0014	0.0017
Methanobrevibacter	0.0112	0.0006	0.0026	0.2696
OTU_136	0.0111	0.0390	0.0401	0.1927
OTU_117	0.0104	0.0000	0.0004	0.2709
OTU_60	0.0057	0.1434	0.0047	0.1175
OTU_143	0.0088	0.0002	0.0206	0.2378
OTU_68	0.2567	0.0013	0.0020	0.0014
OTU_105	0.0120	0.0032	0.0000	0.2398
OTU_359	0.0990	0.0730	0.0537	0.0279
OTU_198	0.2448	0.0015	0.0012	0.0029
Solobacterium	0.0154	0.0332	0.0011	0.1801
Nocardioides	0.2153	0.0008	0.0010	0.0018
OTU_472	0.1717	0.0004	0.0043	0.0228
Butyrivibrio	0.0175	0.0004	0.0011	0.1692
OTU_86	0.1802	0.0011	0.0011	0.0036
Azospirillum	0.1811	0.0011	0.0002	0.0032
Haemophilus	0.0993	0.0014	0.0414	0.0414
Megamonas	0.0013	0.0017	0.1439	0.0351
Phenylobacterium	0.1740	0.0005	0.0023	0.0016
OTU_127	0.0171	0.0002	0.0005	0.1538
Mogibacterium	0.0152	0.0028	0.0005	0.1495
Leuconostoc	0.0007	0.0598	0.1059	0.0000
Enhydrobacter	0.1578	0.0010	0.0014	0.0010
OTU_154	0.0153	0.0001	0.0005	0.1452
OTU_534	0.0040	0.0000	0.0100	0.1459
Atopobium	0.0037	0.0001	0.0008	0.1544
Alistipes	0.0002	0.0009	0.0018	0.1557
OTU_208	0.0196	0.0000	0.0000	0.1378
Pseudobutyrivibrio	0.0045	0.0004	0.0016	0.1410
Rothia	0.0475	0.0403	0.0455	0.0120
OTU_152	0.0037	0.0002	0.0087	0.1324
OTU_438	0.1434	0.0004	0.0000	0.0000
OTU_126	0.0010	0.0026	0.0016	0.1361
Paucimonas	0.1352	0.0008	0.0013	0.0005
OTU_455	0.0059	0.0219	0.0174	0.0923
RC9 gut group	0.0046	0.0004	0.0000	0.1317
OTU_528	0.0132	0.0000	0.0012	0.1218
Anaerotruncus	0.0028	0.0000	0.0029	0.1272
OTU_339	0.0062	0.0001	0.0000	0.1264
Cupriavidus	0.1258	0.0005	0.0011	0.0015
OTU_306	0.0025	0.0804	0.0005	0.0448
OTU_383	0.0002	0.0001	0.0000	0.1238
Deinococcus	0.1153	0.0006	0.0018	0.0014
OTU_177	0.0139	0.0003	0.0012	0.1013
OTU_285	0.0025	0.0034	0.0217	0.0866
Moraxella	0.1097	0.0007	0.0000	0.0017
OTU_249	0.0022	0.0004	0.0018	0.1055
OTU_319	0.0079	0.0000	0.0018	0.0996
Alkanindiges	0.1032	0.0004	0.0002	0.0022
OTU_199	0.0071	0.0000	0.0022	0.0957
OTU_124	0.0998	0.0002	0.0010	0.0007
Microvirga	0.0978	0.0003	0.0002	0.0022
OTU_414	0.0993	0.0005	0.0000	0.0001
OTU_377	0.0962	0.0004	0.0007	0.0019
OTU_384	0.0969	0.0002	0.0004	0.0012
OTU_594	0.0176	0.0428	0.0291	0.0091
Neisseria	0.0965	0.0003	0.0000	0.0014
OTU_174	0.0266	0.0181	0.0220	0.0308
OTU_330	0.0000	0.0071	0.0789	0.0112
OTU_156	0.0004	0.0267	0.0132	0.0561
OTU_588	0.0952	0.0000	0.0000	0.0004
OTU_318	0.0086	0.0000	0.0014	0.0856
Rheinheimera	0.0902	0.0006	0.0000	0.0011
Finegoldia	0.0757	0.0083	0.0025	0.0051

OTU: Operational taxonomic unit
Bacterial proportions >0.5% are highlighted in bold

Supplementary Table S3. Faecal bacterial proportions observed from maternal and infant faecal specimens (n=305) included in the study (continued)

	% of bacteria			
	**Infants at birth (n=107)**	**Infants at 4 to 12 weeks (n=72)**	**Infants at 20-28 weeks (n=36)**	**Mothers at birth (n=90)**
Paracoccus	0.0879	0.0006	0.0000	0.0013
OTU_157	0.0867	0.0003	0.0008	0.0008
Phascolarctobacterium	0.0158	0.0011	0.0031	0.0687
Rubellimicrobium	0.0858	0.0001	0.0002	0.0000
Mitsuokella	0.0035	0.0009	0.0031	0.0784
Bradyrhizobium	0.0828	0.0002	0.0000	0.0018
OTU_195	0.0810	0.0002	0.0000	0.0010
Methylobacterium	0.0789	0.0002	0.0000	0.0004
Roseomonas	0.0756	0.0004	0.0004	0.0008
Salinarimonas	0.0756	0.0000	0.0000	0.0005
OTU_363	0.0042	0.0022	0.0550	0.0133
OTU_529	0.0327	0.0244	0.0148	0.0026
OTU_192	0.0047	0.0000	0.0000	0.0696
OTU_450	0.0062	0.0002	0.0032	0.0636
Flavobacterium	0.0688	0.0005	0.0006	0.0020
OTU_151	0.0209	0.0135	0.0187	0.0188
OTU_355	0.0015	0.0000	0.0000	0.0610
Flavonifractor	0.0008	0.0113	0.0309	0.0186
OTU_607	0.0009	0.0000	0.0109	0.0468
OTU_236	0.0161	0.0127	0.0147	0.0148
Parasutterella	0.0000	0.0002	0.0137	0.0437
Oribacterium	0.0076	0.0002	0.0000	0.0495
Bilophila	0.0003	0.0018	0.0010	0.0532
Vogesella	0.0550	0.0000	0.0000	0.0008
OTU_569	0.0016	0.0006	0.0000	0.0534
OTU_589	0.0491	0.0007	0.0014	0.0010
OTU_271	0.0030	0.0000	0.0007	0.0484
OTU_439	0.0001	0.0000	0.0509	0.0008
OTU_171	0.0499	0.0001	0.0000	0.0015
OTU_184	0.0149	0.0103	0.0104	0.0147
OTU_601	0.0021	0.0000	0.0015	0.0466
OTU_575	0.0034	0.0000	0.0013	0.0447
OTU_182	0.0122	0.0099	0.0124	0.0138
OTU_353	0.0017	0.0122	0.0054	0.0287
OTU_185	0.0140	0.0111	0.0056	0.0160
OTU_161	0.0131	0.0096	0.0084	0.0153
OTU_411	0.0000	0.0000	0.0005	0.0458
OTU_303	0.0108	0.0085	0.0122	0.0145
OTU_290	0.0141	0.0079	0.0092	0.0133
Azorhizobium	0.0433	0.0000	0.0006	0.0003
OTU_204	0.0092	0.0102	0.0077	0.0165
Methanosphaera	0.0025	0.0000	0.0007	0.0399
OTU_406	0.0000	0.0003	0.0005	0.0413
Cloacibacterium	0.0398	0.0000	0.0009	0.0007
Actinomyces	0.0042	0.0057	0.0143	0.0171
OTU_441	0.0012	0.0000	0.0000	0.0401
Eggerthella	0.0002	0.0066	0.0323	0.0018
OTU_613	0.0160	0.0000	0.0000	0.0244
Dyadobacter	0.0394	0.0000	0.0000	0.0000
OTU_287	0.0117	0.0055	0.0062	0.0158
OTU_556	0.0015	0.0001	0.0000	0.0369
Sutterella	0.0009	0.0066	0.0245	0.0063
Bosea	0.0373	0.0000	0.0000	0.0002
Novispirillum	0.0351	0.0000	0.0008	0.0015
OTU_190	0.0081	0.0084	0.0093	0.0112
OTU_314	0.0111	0.0079	0.0078	0.0099
Fusobacterium	0.0106	0.0000	0.0000	0.0255
OTU_401	0.0031	0.0000	0.0000	0.0329
OTU_417	0.0342	0.0000	0.0000	0.0006
OTU_252	0.0344	0.0000	0.0000	0.0001
OTU_440	0.0000	0.0000	0.0000	0.0329
OTU_327	0.0118	0.0041	0.0070	0.0097
OTU_612	0.0000	0.0000	0.0124	0.0199
OTU_216	0.0001	0.0099	0.0057	0.0163
OTU_586	0.0012	0.0000	0.0000	0.0307

OTU: Operational taxonomic unit

Bacterial proportions >0.5% are highlighted in bold

Supplementary Table S3. Faecal bacterial proportions observed from maternal and infant faecal specimens (n=305) included in the study (continued)

	% of bacteria			
	**Infants at birth (n=107)**	**Infants at 4 to 12 weeks (n=72)**	**Infants at 20-28 weeks (n=36)**	**Mothers at birth (n=90)**
Anaerococcus	0.0293	0.0004	0.0000	0.0017
Helicobacter	0.0001	0.0000	0.0307	0.0000
OTU_288	0.0001	0.0000	0.0091	0.0216
OTU_465	0.0003	0.0000	0.0000	0.0304
OTU_545	0.0001	0.0001	0.0000	0.0300
Slackia	0.0018	0.0025	0.0008	0.0249
OTU_244	0.0299	0.0000	0.0000	0.0000
OTU_284	0.0019	0.0000	0.0029	0.0249
Bdellovibrio	0.0291	0.0000	0.0000	0.0006
OTU_220	0.0011	0.0000	0.0000	0.0277
Eubacterium	0.0004	0.0107	0.0160	0.0016
OTU_265	0.0062	0.0052	0.0087	0.0087
OTU_278	0.0069	0.0050	0.0066	0.0099
OTU_337	0.0271	0.0000	0.0000	0.0009
OTU_539	0.0007	0.0004	0.0000	0.0269
Peptoniphilus	0.0191	0.0028	0.0020	0.0037
OTU_610	0.0012	0.0002	0.0000	0.0242
Achromobacter	0.0252	0.0002	0.0000	0.0002
OTU_617	0.0005	0.0000	0.0065	0.0185
OTU_300	0.0250	0.0000	0.0000	0.0000
Geodermatophilus	0.0240	0.0000	0.0000	0.0005
OTU_341	0.0039	0.0047	0.0088	0.0068
Acidaminococcus	0.0002	0.0066	0.0000	0.0172
OTU_371	0.0240	0.0000	0.0000	0.0000
OTU_234	0.0076	0.0043	0.0078	0.0040
Chryseomicrobium	0.0230	0.0000	0.0000	0.0002
OTU_302	0.0219	0.0003	0.0004	0.0000
OTU_259	0.0055	0.0034	0.0062	0.0073
OTU_548	0.0215	0.0000	0.0005	0.0003
Sediminibacterium	0.0217	0.0002	0.0000	0.0005
OTU_280	0.0058	0.0050	0.0073	0.0041
OTU_564	0.0053	0.0000	0.0000	0.0169
OTU_561	0.0004	0.0000	0.0000	0.0215
Peredibacter	0.0212	0.0000	0.0000	0.0004
OTU_350	0.0052	0.0038	0.0049	0.0076
OTU_515	0.0000	0.0000	0.0029	0.0185
Tepidiphilus	0.0206	0.0000	0.0000	0.0004
OTU_245	0.0195	0.0006	0.0000	0.0006
OTU_381	0.0000	0.0000	0.0000	0.0204
OTU_263	0.0043	0.0032	0.0045	0.0082
OTU_257	0.0201	0.0001	0.0000	0.0000
OTU_538	0.0023	0.0000	0.0000	0.0178
OTU_267	0.0008	0.0000	0.0000	0.0181
OTU_574	0.0184	0.0000	0.0000	0.0002
Novosphingobium	0.0180	0.0000	0.0000	0.0004
Marvinbryantia	0.0014	0.0000	0.0000	0.0170
OTU_332	0.0059	0.0043	0.0034	0.0047
OTU_223	0.0176	0.0000	0.0000	0.0005
OTU_313	0.0059	0.0021	0.0044	0.0057
OTU_351	0.0069	0.0026	0.0022	0.0060
Epulopiscium	0.0000	0.0059	0.0108	0.0007
OTU_308	0.0163	0.0000	0.0000	0.0008
Aeromicrobium	0.0159	0.0000	0.0000	0.0004
Enterorhabdus	0.0014	0.0073	0.0011	0.0061
OTU_364	0.0042	0.0013	0.0036	0.0065
Flavisolibacter	0.0148	0.0000	0.0004	0.0003
Aliidiomarina	0.0152	0.0003	0.0000	0.0000
OTU_577	0.0005	0.0000	0.0000	0.0150
Dietzia	0.0136	0.0000	0.0000	0.0009
OTU_289	0.0045	0.0026	0.0029	0.0044
Vibrio	0.0135	0.0000	0.0000	0.0000
OTU_522	0.0124	0.0006	0.0000	0.0000
OTU_452	0.0045	0.0026	0.0028	0.0025
Parasegetibacter	0.0117	0.0001	0.0000	0.0000
OTU_398	0.0026	0.0023	0.0041	0.0021
Rhodocista	0.0100	0.0000	0.0000	0.0000

OTU: Operational taxonomic unit

Bacterial proportions >0.5% are highlighted in bold

Supplementary Table S3. Faecal bacterial proportions observed from maternal and infant faecal specimens (n=305) included in the study (continued)

	% of bacteria			
	**Infants at birth (n=107)**	**Infants at 4 to 12 weeks (n=72)**	**Infants at 20-28 weeks (n=36)**	**Mothers at birth (n=90)**
Sphingopyxis	0.0092	0.0000	0.0000	0.0000
Stenotrophomonas	0.0079	0.0000	0.0007	0.0003
OTU_358	0.0038	0.0029	0.0008	0.0012
Mycoplasma	0.0086	0.0000	0.0000	0.0000
Brachybacterium	0.0084	0.0000	0.0000	0.0000
OTU_382	0.0005	0.0000	0.0000	0.0079
uncultured gamma proteobacterium	0.0082	0.0000	0.0000	0.0000
OTU_514	0.0080	0.0000	0.0000	0.0000
Adhaeribacter	0.0080	0.0000	0.0000	0.0000
Campylobacter	0.0076	0.0003	0.0000	0.0000
uncultured alpha proteobacterium	0.0078	0.0000	0.0000	0.0000
Facklamia	0.0072	0.0000	0.0000	0.0000
OTU_473	0.0068	0.0000	0.0000	0.0000
Skermanella	0.0067	0.0000	0.0000	0.0000
OTU_374	0.0000	0.0054	0.0000	0.0002
Micrococcus	0.0051	0.0000	0.0000	0.0003
Simiduia	0.0048	0.0000	0.0000	0.0000
OTU_210	0.0044	0.0000	0.0000	0.0000
OTU_568	0.0044	0.0000	0.0000	0.0000
OTU_311	0.0040	0.0000	0.0000	0.0000
Triticum aestivum (bread wheat)	0.0034	0.0000	0.0005	0.0000
uncultured actinobacterium	0.0036	0.0000	0.0000	0.0000
Cellvibrio	0.0033	0.0000	0.0000	0.0000
Mycobacterium	0.0033	0.0000	0.0000	0.0000
OTU_488	0.0031	0.0000	0.0000	0.0000
OTU_373	0.0027	0.0002	0.0000	0.0000
Arthrobacter	0.0024	0.0000	0.0000	0.0000
Tahibacter	0.0024	0.0000	0.0000	0.0000
OTU_576	0.0022	0.0000	0.0000	0.0000
OTU_542	0.0017	0.0000	0.0000	0.0000
Marmoricola	0.0016	0.0000	0.0000	0.0000
Trueperella	0.0015	0.0000	0.0000	0.0000
OTU_602	0.0014	0.0000	0.0000	0.0000
OTU_587	0.0014	0.0000	0.0000	0.0000
OTU_552	0.0013	0.0000	0.0000	0.0000
OTU_468	0.0013	0.0000	0.0000	0.0000
uncultured soil bacterium	0.0006	0.0000	0.0000	0.0000
OTU_597	0.0005	0.0000	0.0000	0.0000
Nannocystis	0.0000	0.0000	0.0000	0.0000

OTU: Operational taxonomic unit
Bacterial proportions >0.5% are highlighted in bold

Supplementary Table S4. Significant differences in faecal bacterial proportions from 36 infants with specimens collected at birth, 4-12 and 20-28 weeks.

	**Infants at birth (n=36) Median (IQR)**	**Infants at 4-12 weeks (n=36) Median (IQR)**	**Infants at 20-28 weeks (n=36) Median (IQR)**	**p-value**
**Phylum Proteobacteria**	**61.4 (3.1-91.7)**	10.1 (1.6-52.9)	6.4 (2.2-21.2)	<0.001
Alphaproteobacteria	-	-	-	-
Caulobacterales	**0.2 (0.1-4.3)**	0.1 (0.0-0.1)	0.1 (0.0-0.1)	0.004
Caulobacteraceae	-	-	-	-
Brevundimonas	**0.1 (0.1-2.5)**	0.0 (0.0-0.0)	0.0 (0.0-0.1)	0.018
OTU_15	**0.0 (0.0-0.1)**	0.0 (0.0-0.0)	0.0 (0.0-0.0)	0.023
OTU_541	**0.0 (0.0-0.7)**	0.0 (0.0-0.0)	0.0 (0.0-0.0)	0.029
Sphingomonadales	**0.0 (0.0-2.0)**	0.0 (0.0-0.0)	0.0 (0.0-0.0)	0.038
Betaproteobacteria	-	-	-	-
Burkholderiales	**0.2 (0.1-21.7)**	0.0 (0.0-0.1)	0.0 (0.00-0.1)	0.031
Comamonadaceae	-	-	-	-
OTU_14	**0.0 (0.0-1.9)**	0.0 (0.0-0.0)	0.0 (0.0-0.0)	0.024
Oxalobacteraceae	-	-	-	-
OTU_18	**0.0 (0.0-0.5)**	0.0 (0.0-0.0)	0.0 (0.0-0.0)	0.024
Gammaproteobacteria	-	-	-	-
aaa34a10	**0.0 (0.0-0.2)**	0.0 (0.0-0.0)	0.0 (0.0-0.0)	0.031
Alteromonadales	**0.1 (0.0-1.6)**	0.0 (0.0-0.1)	0.0 (0.0-0.0)	0.010
Shewanellaceae	-	-	-	-
Shewanella	**0.1 (0.0-1.6)**	0.0 (0.0-0.1)	0.0 (0.0-0.0)	0.024
Pseudomonadales	**0.3 (0.1-15.6)**	0.1 (0.0-0.1)	0.1 (0.0-0.1)	0.018
Moraxellaceae	-	-	-	-
Acinetobacter	**0.2 (0.1-7.6)**	0.0 (0.0-0.1)	0.0 (0.0-0.1)	0.026
**Phylum Firmicutes**	-	-	-	-
Bacilli	1.5 (0.5-3.1)	**9.3 (5.0-24.2)**	8.8 (3.3-10.7)	0.005
Bacillales	**0.1 (0.0-0.3)**	0.0 (0.0-0.0)	0.0 (0.0-0.1)	<0.005*
Staphylococcaceae	**0.0 (0.0-0.1)**	0.0 (0.0-0.0)	0.0 (0.0-0.1)	<0.001×
Staphylococcus	**0.0 (0.0-0.1)**	0.0 (0.0-0.0)	0.0 (0.0-0.1)	0.002×
Lactobacillales	1.3 (0.4-2.8)	**9.3 (4.9-24.2)**	8.8 (3.2-10.7)	0.001
**Phylum Actinobacteria**	8.5 (2.9-16.7)	**65.3 (9.3-81.8)**	50.0 (20.5-67.3)	<0.001*
Actinobacteria	6.3 (2.2-14.6)	**62.7 (4.9-77.9)**	47.1 (19.8-67.3)	<0.001*
Bifidobacteriales	0.9 (0.3-9.6)	**62.6 (4.7-77.9)**	47.1 (19.8-67.3)	<0.001*
Bifidobacteriaceae	0.9 (0.3-9.6)	**62.6 (4.7-77.9)**	47.1 (19.7-67.2)	<0.001*
Bifidobacterium	0.9 (0.3-9.6)	**62.6 (4.7-77.9)**	47.1 (19.7-67.2)	<0.002*
Frankiales	**0.1 (0.0-1.0)**	0.0 (0.0-0.0)	0.0 (0.0-0.0)	0.031

Bacterial proportions are rounded to one decimal point. Larger proportions of bacterial taxa are highlighted in bold. All p-values represent significant differences in bacterial proportions from meconium specimens and faecal specimens collected at 20-28 weeks. Asterisks show significant differences between bacterial proportions observed from meconium specimens and specimens collected at 4-12 weeks of life. P-values denoted by x-symbols only represent significant differences between faecal specimens collected at 4-12 weeks and 20-28 weeks of life. IQR: Interquartile range; OTU: Operational taxonomic unit

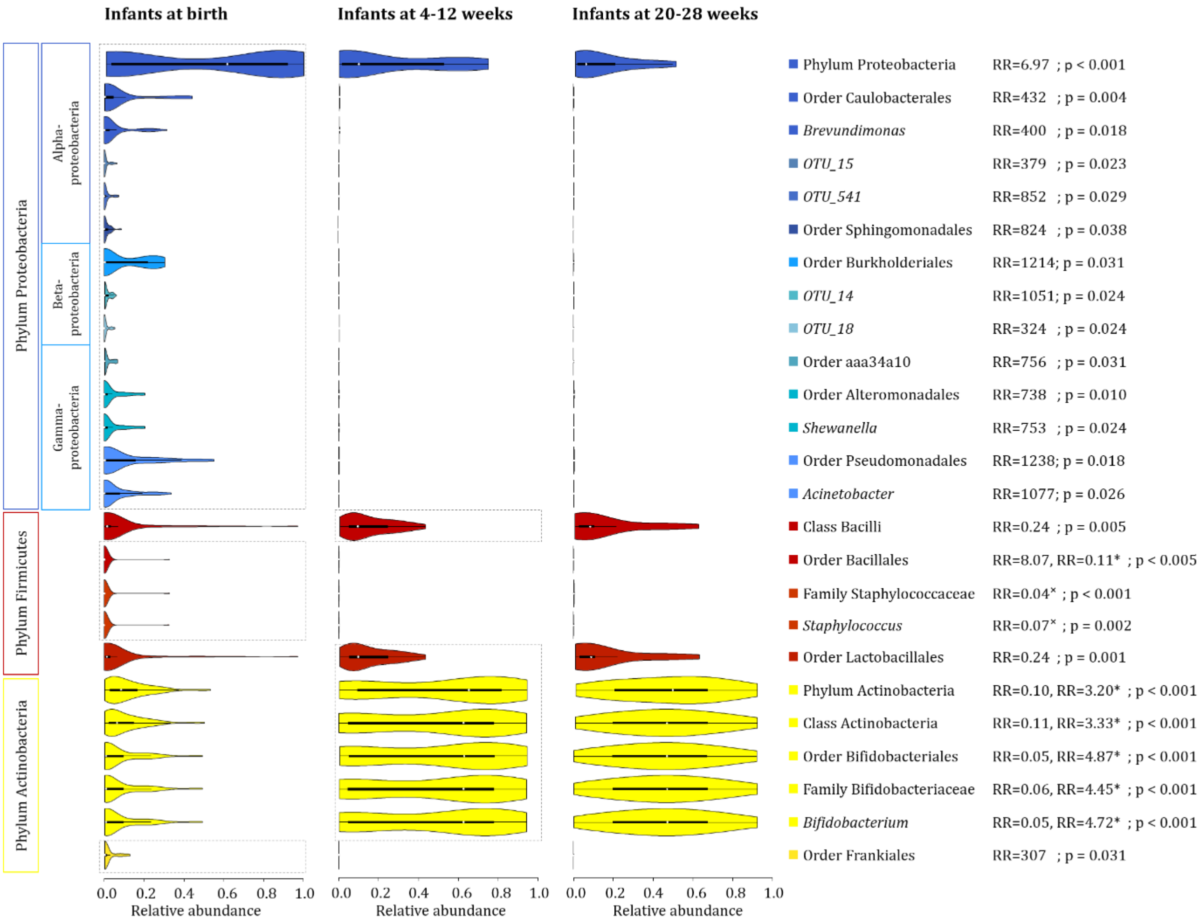

Supplementary Figure S2. Violin plots showing significantly different faecal bacterial profiles when comparing specimens from 36 infants collected at birth, 4-12 and 20-28 weeks

P-values and rate ratios (RR) are summarised for each significant taxon. Unique operational taxonomic unit (OTU) numbers are assigned to each unclassified taxon.

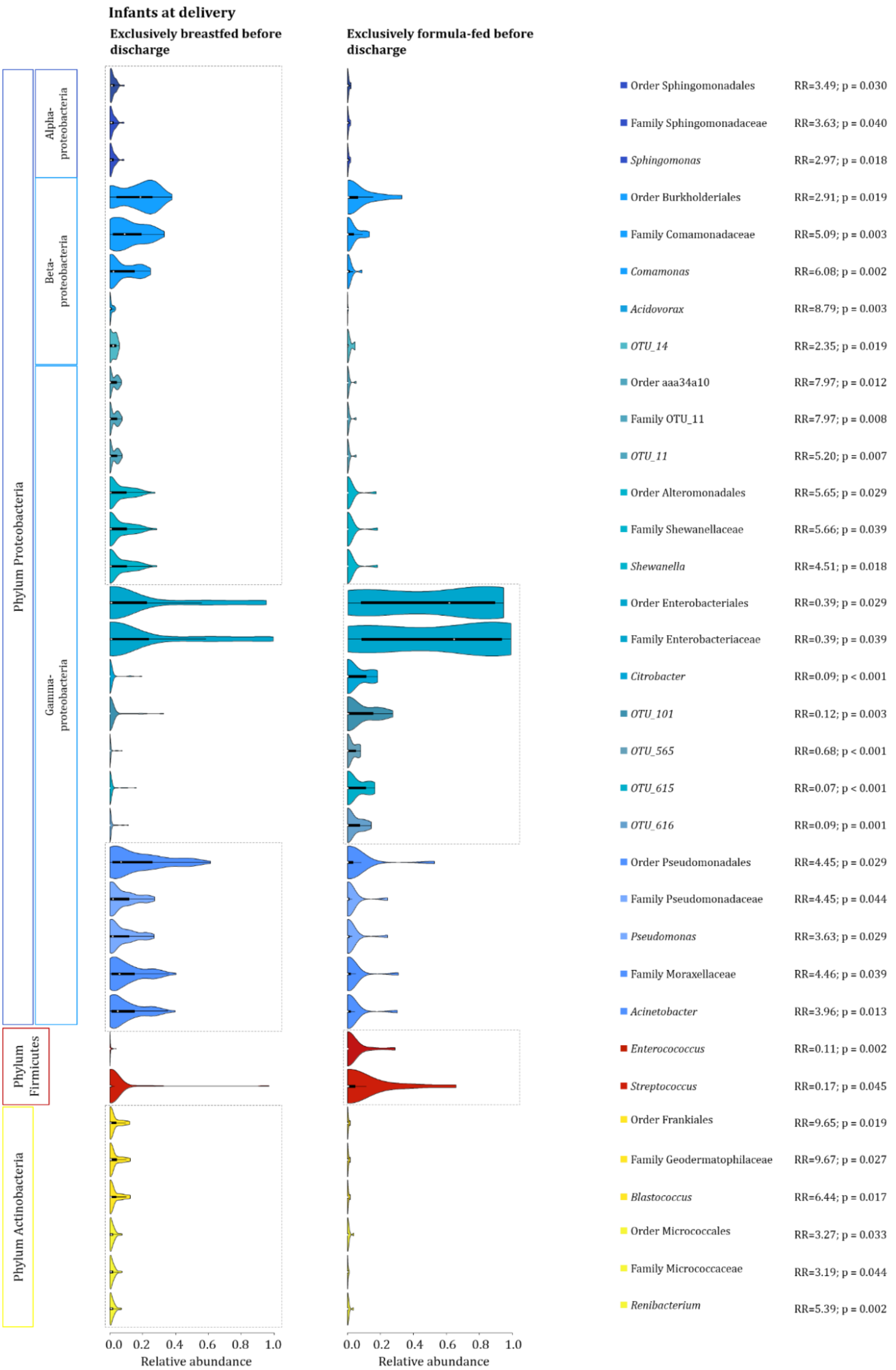

Supplementary Figure S3. Violin plots showing significantly different faecal bacterial profiles from meconium collected at birth in relation to feeding (n=103)

P-values and rate ratios (RR) are summarised for each significant taxon. Unique operational taxonomic unit (OTU) numbers are assigned to each unclassified taxon.

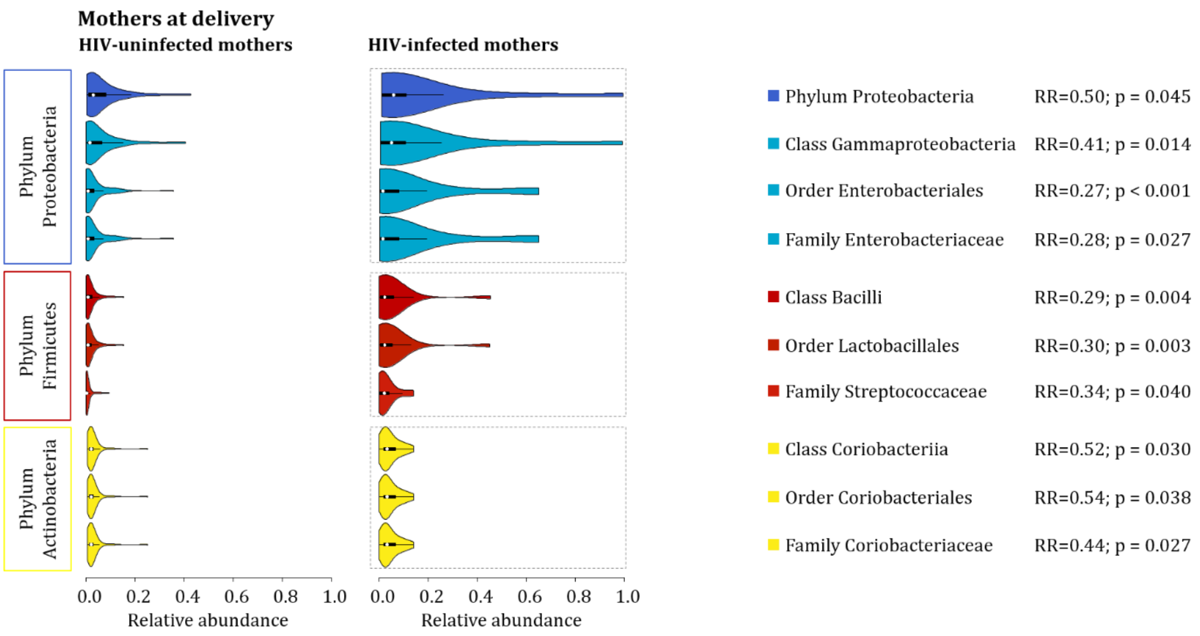

Supplementary Figure S4. Violin plots showing significantly different faecal bacterial profiles from maternal faecal specimens (n=90) collected at delivery in relation to maternal HIV-infection status

P-values and rate ratios (RR) are summarised for each significant taxon.

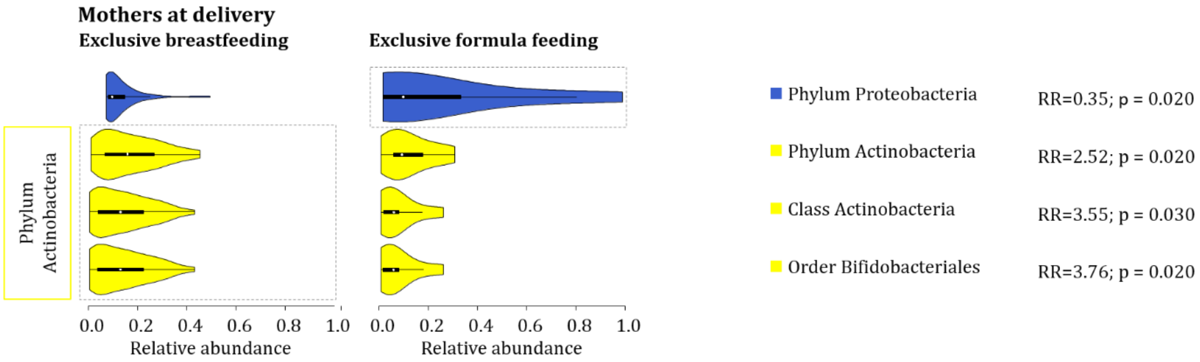

Supplementary Figure S5. Violin plots showing significantly different faecal bacterial profiles from maternal faecal specimens collected at birth (n=86).

P-values and rate ratios (RR) are summarised for each significant taxon.

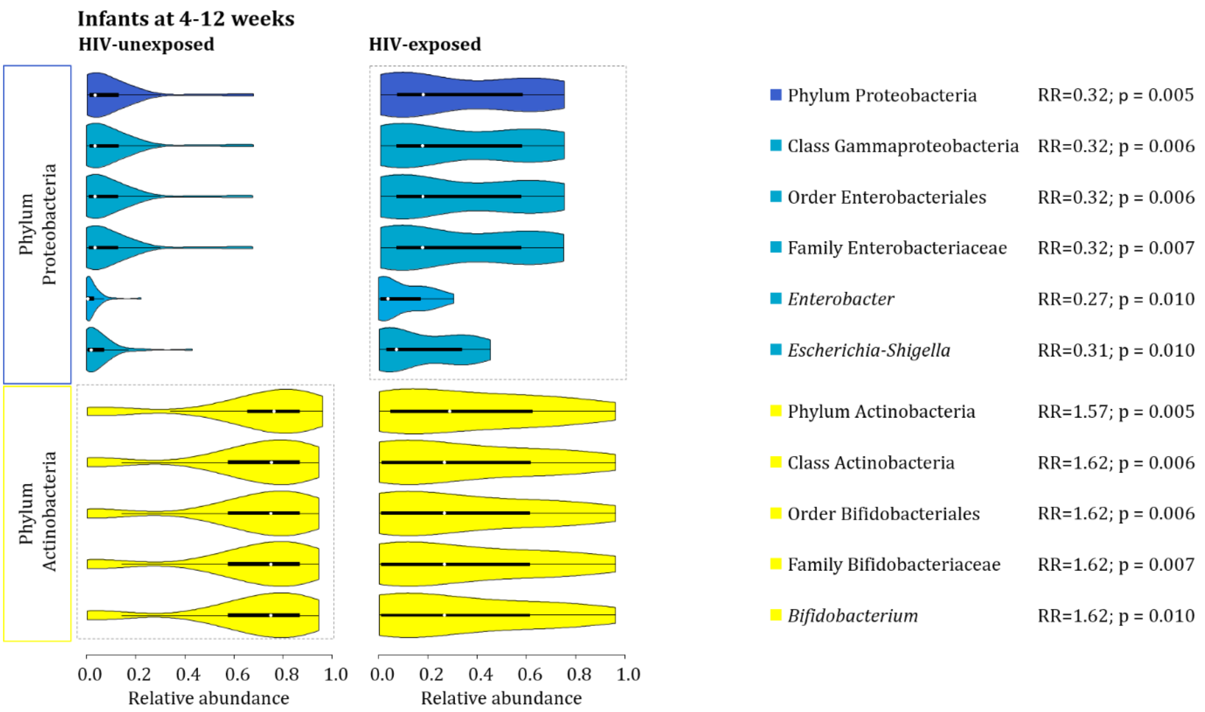

Supplementary Figure S6. Violin plots showing significantly different faecal bacterial profiles from infant faecal specimens collected at 4-12 weeks (n=72) in relation to HIV-exposure. P-values and rate ratios (RR) are summarised for each significant taxon.

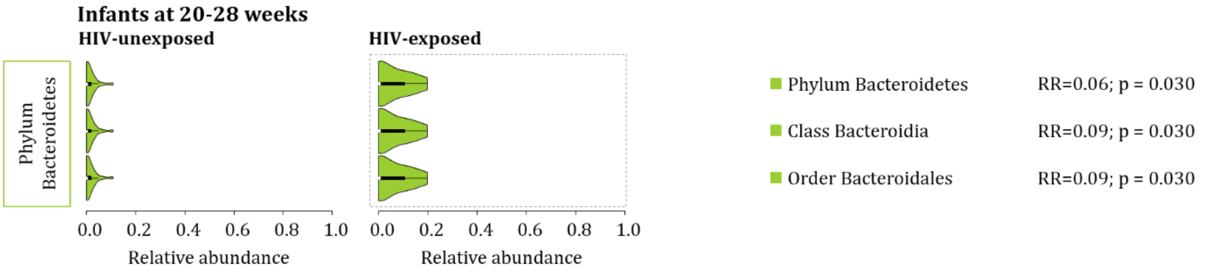

Supplementary Figure S7. Violin plots showing significantly different faecal bacterial profiles from infant faecal specimens collected at 20-28 weeks (n=36) in relation to HIV-exposure. P-values and rate ratios (RR) are summarised for each significant taxon.

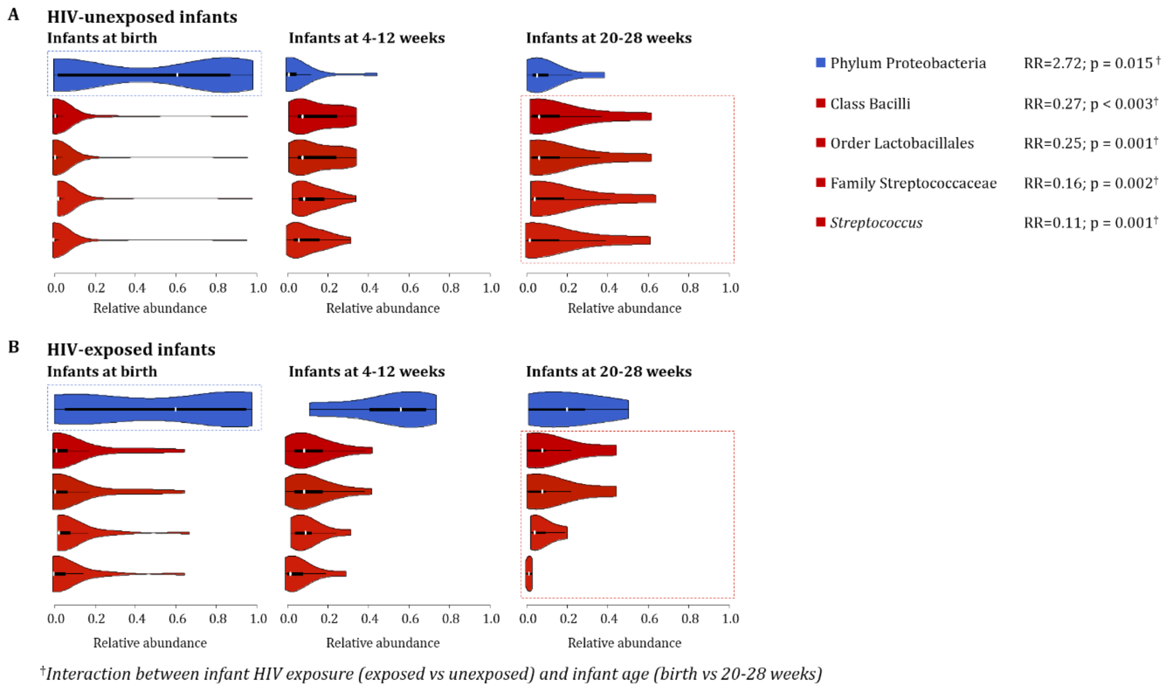

Supplementary Figure S8. Violin plots showing the effect of HIV-exposure on changes in infant faecal bacterial profiles measured over time.

P-values and rate ratios (RR) are summarised for each significant taxon.

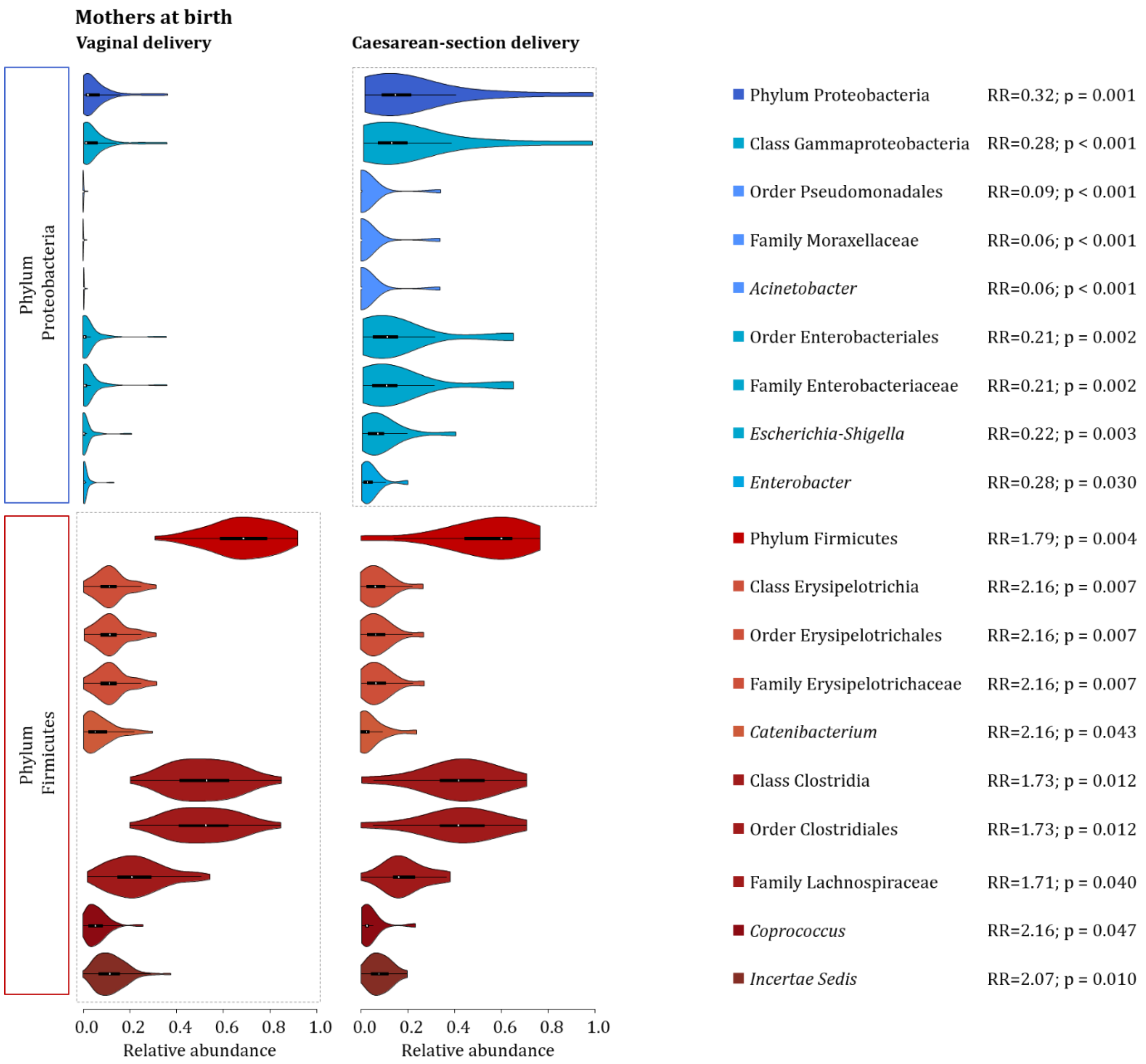

Supplementary Figure S9. Violin plots showing significantly different faecal bacterial profiles from maternal faecal specimens collected at birth in relation to mode of delivery (n=90).
P-values and rate ratios (RR) are summarised for each significant taxon.

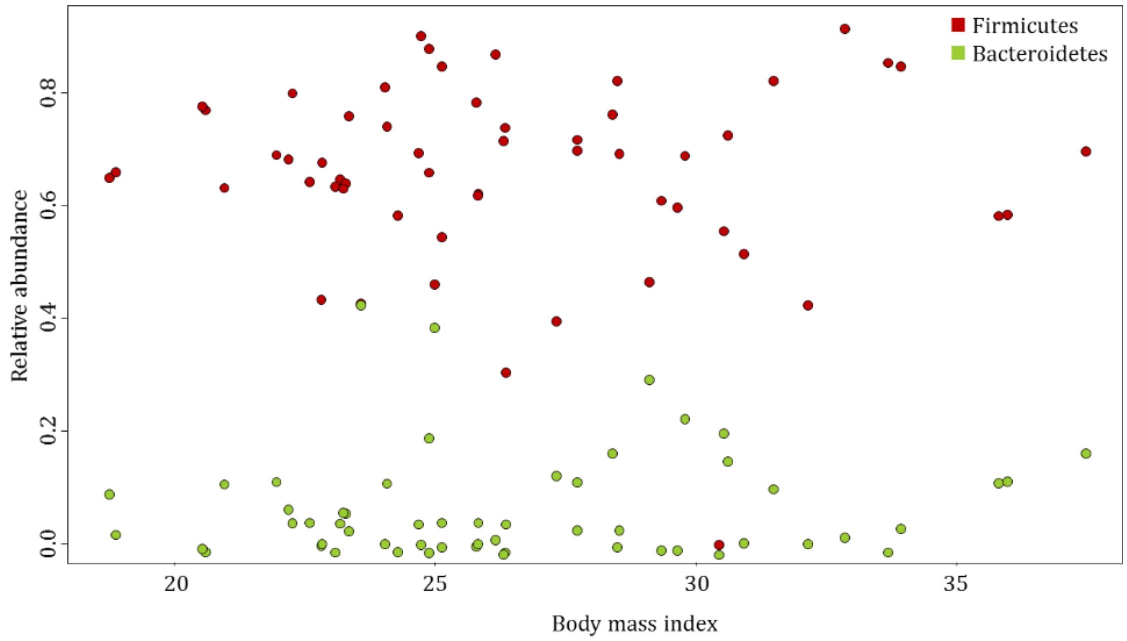

Supplementary Figure S10. Relative abundances of Bacteroidetes and Firmicutes observed for each of the maternal participants under study in relation to their body mass index.

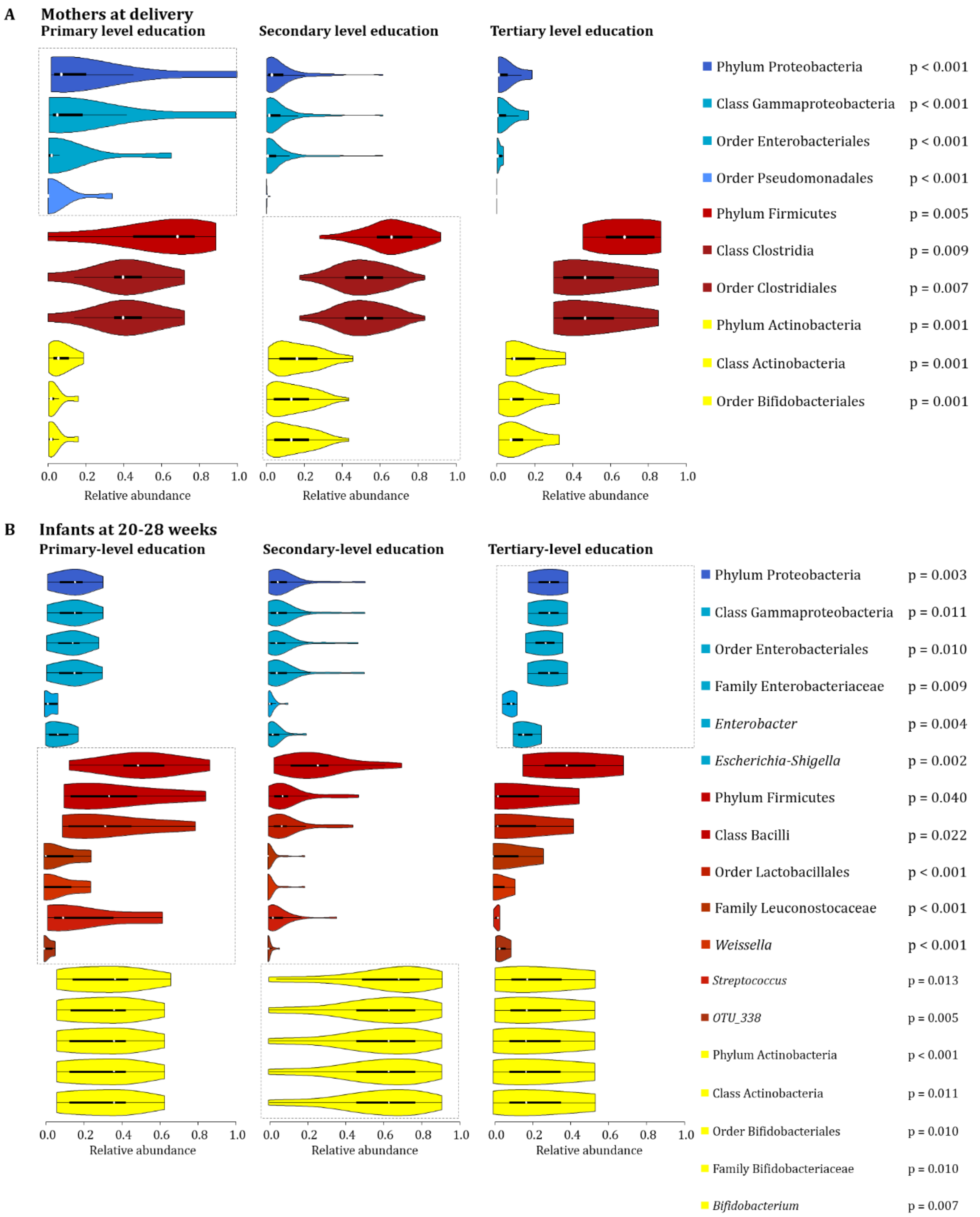

Supplementary Figure S11. Violin plots showing significantly different faecal bacterial profiles from A) maternal faecal specimens collected at birth (n=90) and B) infant faecal specimens collected at 20-28 weeks (n=36) in relation to maternal education.

P-values are summarised for each significant taxon. Unique operational taxonomic unit (OTU) numbers are assigned to each unclassified taxon.

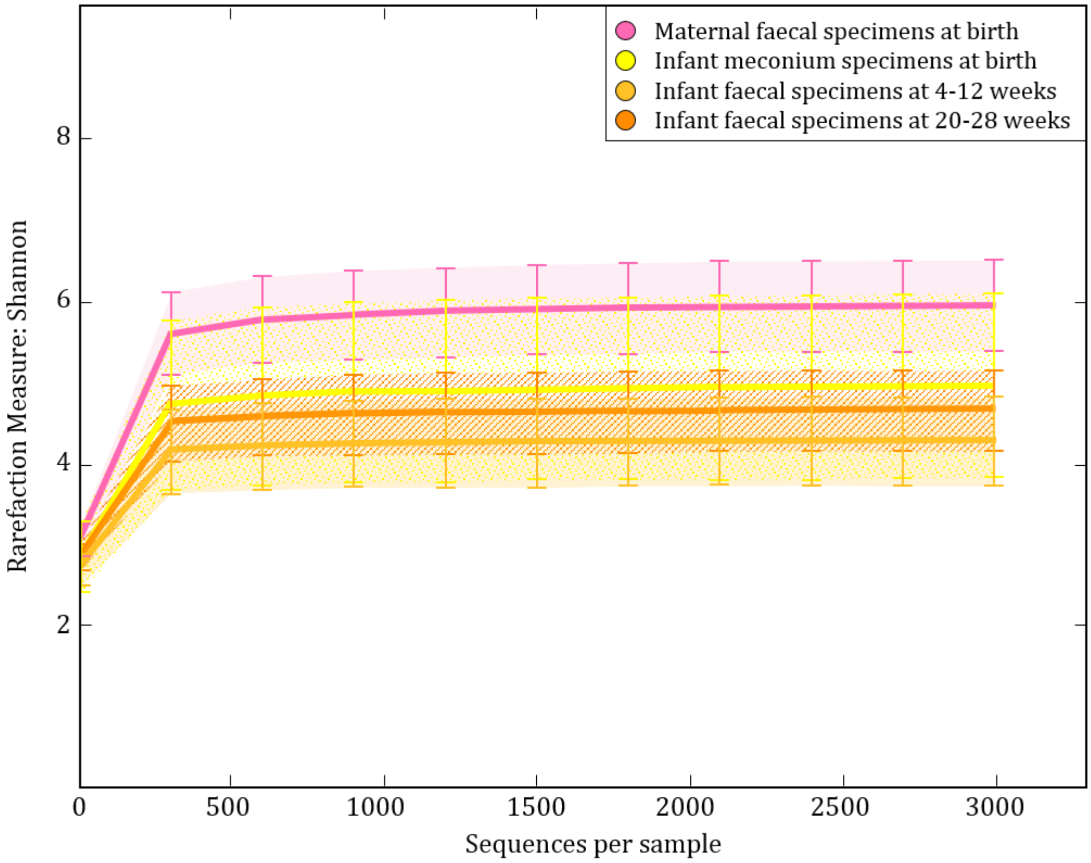

Supplementary Figure S12. 16S rRNA sequencing data output
Rarefaction curve shows the alpha diversity against the number of reads remaining following the removal of contaminants.

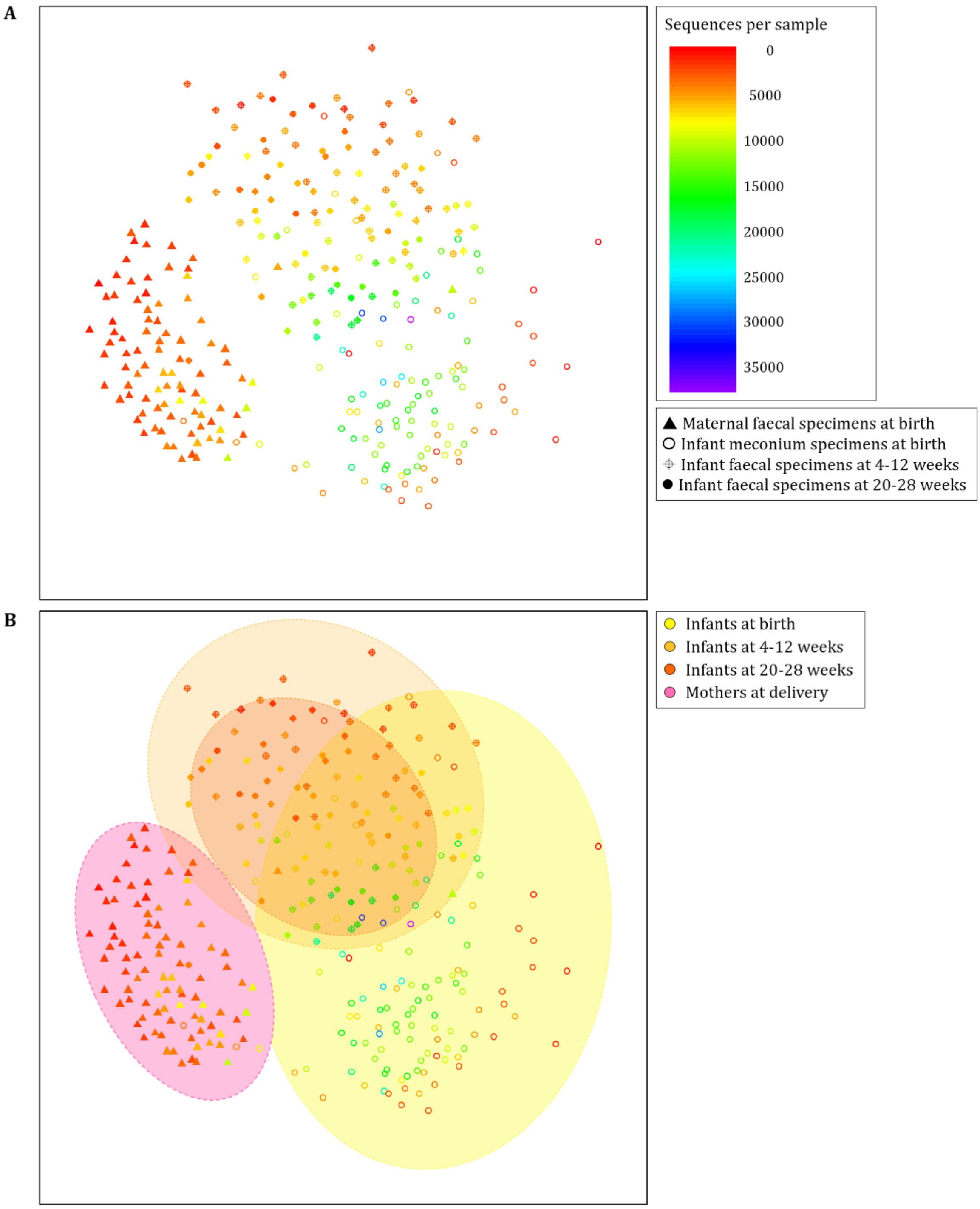

Supplementary Figure S13. Multidimensional scaling (MDS) of samples in relation to their sequencing depth.

A) An increase in sequencing depth is indicated by a range of colours. The red spectrum of colours represents low sequencing depths, while the blue spectrum represents high sequencing depth. Beta diversity is highest among samples with lower sequencing depth. Samples from each participant group in the MDS plot are distinguished using different symbols. B) Samples from each participant group cluster together.

A

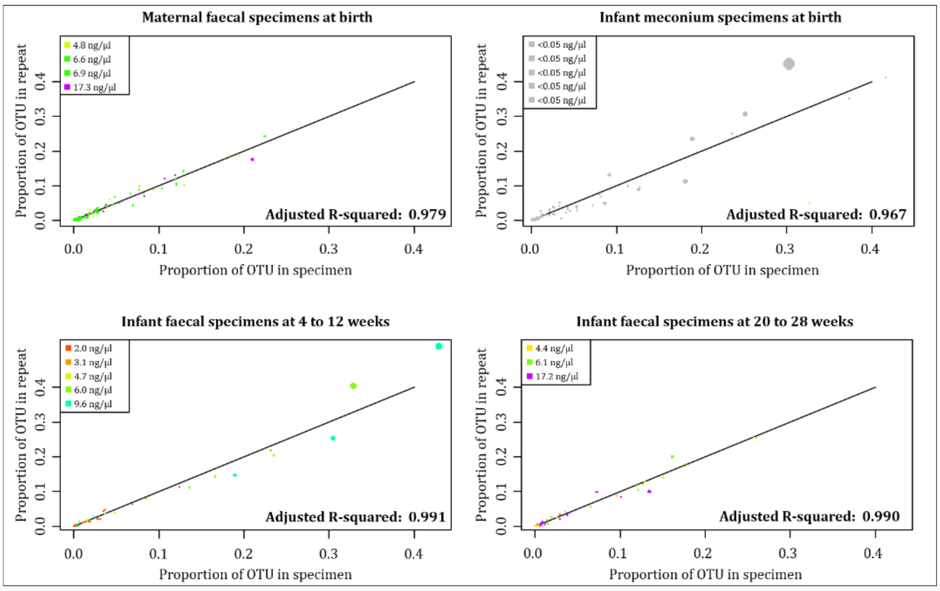

B

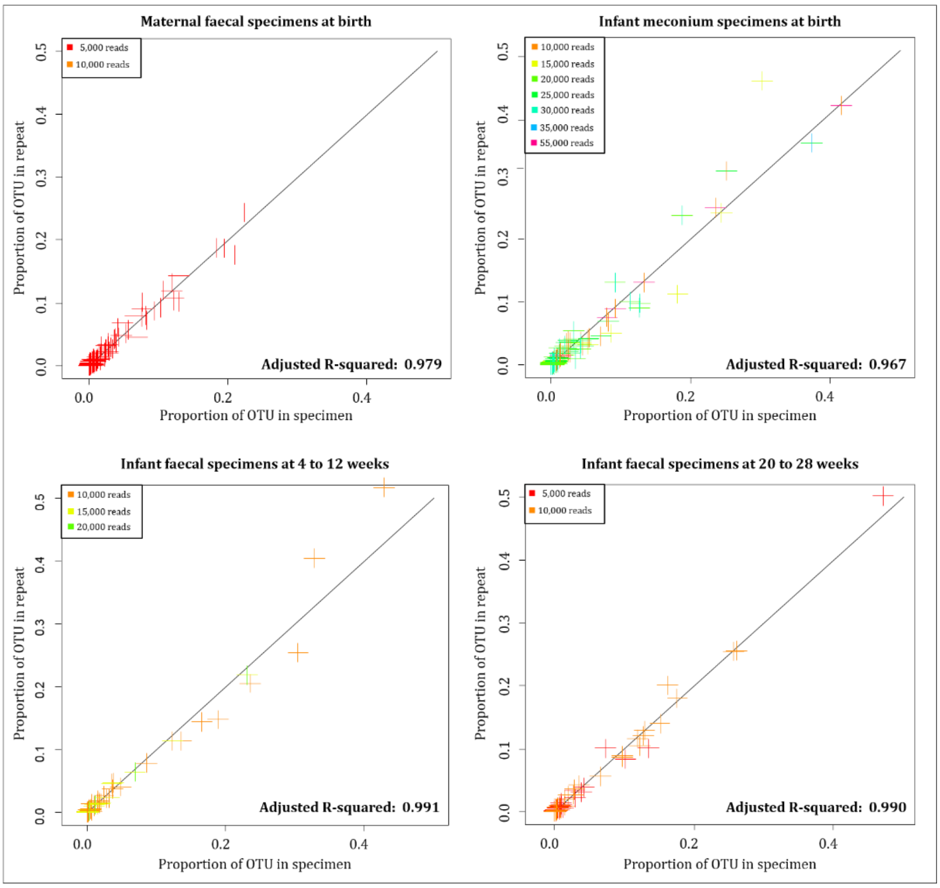

Supplementary Figure S14. 16S rRNA sequencing reproducibility in relation to template concentration and sequencing depth

Scatter plots represent the proportion of each operational taxonomic unit (OTU) detected from the 17 faecal specimens (X-axis) and their technical repeats (Y-axis). The distance between each point on the plot and the diagonal line measures the variation between the specimens processed in duplicate – the larger the distance, the more dissimilar the proportions of the respective OTU between the technical repeats. A) Nucleic acid concentrations of each specimen are indicated using a colour scale. Colours range from grey (< 0.05 ng/µl) to purple (representing high nucleic acid concentrations). B) The number or reads sequenced from each specimen are shown using a colour scale. Colours range from red, representing the lowest spectrum of sequencing depth, to pink, representing the highest spectrum.
